# From iron coordination compounds to metal oxide nanoparticles

**DOI:** 10.3762/bjnano.7.198

**Published:** 2016-12-28

**Authors:** Mihail Iacob, Carmen Racles, Codrin Tugui, George Stiubianu, Adrian Bele, Liviu Sacarescu, Daniel Timpu, Maria Cazacu

**Affiliations:** 1 Inorganic Polymers Department, “Petru Poni” Institute of Macromolecular Chemistry, Aleea Gr. Ghica Voda 41A, Iasi, 700487, Romania

**Keywords:** iron coordination compounds, mixed oxide nanoparticles, morphology control, nanoparticle shape control, optimization procedure

## Abstract

Various types, shapes and sizes of iron oxide nanoparticles were obtained depending on the nature of the precursor, preparation method and reaction conditions. The mixed valence trinuclear iron acetate, [Fe_2_^III^Fe^II^O(CH_3_COO)_6_(H_2_O)_3_]·2H_2_O (FeAc1), μ_3_-oxo trinuclear iron(III) acetate, [Fe_3_O(CH_3_COO)_6_(H_2_O)_3_]NO_3_∙4H_2_O (FeAc2), iron furoate, [Fe_3_O(C_4_H_3_OCOO)_6_(CH_3_OH)_3_]NO_3_∙2CH_3_OH (FeF), iron chromium furoate, FeCr_2_O(C_4_H_3_OCOO)_6_(CH_3_OH)_3_]NO_3_∙2CH_3_OH (FeCrF), and an iron complex with an original macromolecular ligand (FePAZ) were used as precursors for the corresponding oxide nanoparticles. Five series of nanoparticle samples were prepared employing either a classical thermal pathway (i.e., thermal decomposition in solution, solvothermal method, dry thermal decomposition/calcination) or using a nonconventional energy source (i.e., microwave or ultrasonic treatment) to convert precursors into iron oxides. The resulting materials were structurally characterized by wide-angle X-ray diffraction and Fourier transform infrared, Raman, energy-dispersive X-ray, and X-ray fluorescence spectroscopies, as well as thermogravimetric analysis. The morphology was characterized by transmission electron microscopy, atomic force microscopy and dynamic light scattering. The parameters were varied within each route to fine tune the size and shape of the formed nanoparticles.

## Introduction

The iron oxide-based materials have been a constant presence in human life throughout mankind’s existence. The first evidence dates from about 100,000 years [1] found in tools for production and storage of ochre (iron oxides and hydroxides) used for painting bodies. When it was found that materials formed from small particles exhibit different properties from their bulk form [2], numerous researchers became interested in discovering new properties and applications. Nanometer-sized iron oxides proved to be of interest in several fields such as medicine, applied physics, chemistry and engineering [3–6]. The interest in iron oxide nanoparticles and their use in an extremely large number of applications is motivated by stability, biocompatibility, magnetic properties and their availability. However, certain applications require a rigorous selection of the nanoparticles by size and shape because these parameters determine the number of surface atoms, which is decisive for their properties. The surface of the nanoparticle increases with decreasing particle size and also depends on its geometry [7]. For a certain type of magnetic nanoparticles, their size and/or geometry define the magnetic transitions. For example, magnetite is ferromagnetic when the particle diameter is larger than 15 nm and superparamagnetic when smaller [8]. Zhen et al. demonstrate that cubic nanoparticles have higher saturation magnetization and T2 relaxation than spherical nanoparticles of the same size [9]. Magnetic nanoparticles with flat surfaces are often used for biomedical applications (e.g., biosensing, hyperthermia and MRI) [10]. In biomedical applications, the morphology of the nanoparticle significantly influences both pharmacokinetics and cell uptake [11]. Nanoparticles are also preferred as fillers for polymers to induce certain properties upon the resulting composites. The effect will be greater as the particle size is reduced and their dimensional ratio is increased, resulting in a higher matrix–filler contact surface [6]. Therefore, establishing methods for preparing nanoparticles of iron oxide with predetermined dimensional characteristics and morphology is an important task for scientists.

Iron oxide nanoparticles can be obtained using chemical, physical or biological methods. Among the best known chemical methods are coprecipitation, thermal decomposition, hydrothermal method, solvothermal method and others [5,12–13]. An important advantage of chemical methods for preparing nanoparticles lies in the use of different stabilizing agents [4–16] which provide a number of advantages such as stability, dispersibility and compatibility with different backgrounds. The attachment of various groups on the surface of nanoparticles [17–18] is useful for broadening the field of applications in medicine or catalysis [5,17]. Although there are currently numerous methods for the preparation of iron oxides nanoparticles, scientists are working to further improve existing methods and are developing new methods. The preparation of iron oxide nanoparticles is a complex process. The main challenges are finding the optimal experimental conditions in order to obtain monodisperse nanoparticles, ensuring reproducibility and scalability of the process, while limiting complex purification steps [5].

As precursors for obtaining iron oxides or mixed oxides thereof with other metals, their salts are most often used. In recent years, particular attention was paid to the use of coordination compounds as precursors, since they offer a number of advantages such as increased solubility, and better control of formed micro- or nanostructures [19]. Most studies in this area relate to the thermal decomposition of metal complexes with ligands such as acetylacetonates, acetates and oleates. Good results in terms of narrow size distribution of nanoparticles are correlated with nucleation and growth stages that take place separately at different temperatures [20–21]. By varying parameters such as the reaction conditions (e.g., time, temperature), the concentration and types of precursors, it is possible to obtain nanoparticles of various, well-defined sizes and shapes.

From the perspective of obtaining mixed oxide nanoparticles, if we refer to iron/chromium oxides, for example, all methods known in the art to obtain such nanoparticles are based on the use of iron and chromium salts in a specific physical relation. In our previous studies we have demonstrated the possibility of obtaining nanoparticles of iron/chromium oxide from mixed metal clusters with preservation of the mass ratio of iron and chromium [22–23].

In the current study, we present various approaches for preparing iron oxide or mixed oxide nanoparticles using metal complexes as precursors with or without stabilizing agents. Some of the precursors were used for the first time as the metal source for the nanoparticles. Besides classical methods, unconventional ones were also used. The manufacturing processes were optimized to achieve the desired structure, shape, size and dispersity of nanoparticles.

## Results and Discussion

In general, the procedure leading to metal oxide materials consists of the thermal decomposition of adequate precursors under different conditions: by classical heating at high temperature in air (calcination) or in solution, in presence of changing ligands or surfactants. Nonconventional energy sources such as ultrasound and microwaves can also be used. In this work we employed all of these procedures for the conversion of original precursors into proper oxide nanoparticles. The working conditions were identified in which nanoparticles with certain composition, size and shape could be obtained.

### Thermal decomposition in solution

We previously reported on procedures to prepare iron oxide nanowires [24] and iron/chromium oxide nanoparticles [23] with a pre-established ratio between metals from bimetallic molecular precursors via a thermal decomposition method. Here we show, for the first time, the use of a mixed-valence trinuclear acetate cluster to obtain ultra-small iron oxide nanoparticles. These have been prepared by one-step procedure consisting of the thermal decomposition of the reaction mixture that contains mixed-valence iron acetate (FeAc1) as the oxide precursor, dodecylamine (DA) and sunflower oil (SO) as stabilizing agents, and trichloroacetic acid (TCAA) as the solvent (Supporting Information File 1, Figure S1). Sunflower oil was used first time by Turta et al. [25] as a natural alternative for a nanoparticle stabilizing agent. The obtained material was dispersed in hexane and filtered. The filtrate was coded as NPT1a and the sample retained by filter was coded NPT1b and these were further characterized separately (Table 1).

**Table 1:** The experimental conditions used for the conversion of iron-based precursors into nanoparticles with different shapes and sizes by thermal decomposition.

Sample	Feed reactants and experimental conditions					Nanoparticle characteristics	
	Precursor	Surfactant	Solvent	*t* (min)	*T* (˚C)	Diameter (nm)	Morphology

NPT1a^a^	FeAc1 (0.6 mmol)	SO (4 mL), DA (10.8 mmol)	TCAA (15.3 mmol)	30	320	3.5	Spherical with some irregularities
NPT1b^b^	FeAc1 (0.6 mmol)	SO (4 mL), DA (10.8 mmol)	TCAA (15.3 mmol)	30	320	70	Various shapes (hexagon, rods, triangles, etc.)
NPT2	FeF (0.9 mmol)	OO (12 mL), HA (24.8 mmol)	TCAA (59.4 mmol)	10	350	78	Cubic
NPT3	FeCrF (0.9 mmol)	OA (6.7 mmol), DA (29.1 mmol)	TCAA (36.7 mmol)	30	320	12.5	Spherical
NPT4	FeAc2 (1.6 mmol)	OA (0.6 mmol), DA (22.7 mmol)	TCAA (25.7 mmol)	30	320	80	Spheres with hair-like structures protruding from the surface at various angles

^a^Nanoparticles passed through the filter paper; ^b^nanoparticles remained on the filter paper.

In the IR spectrum of NPT1a (Supporting Information File 1, Figure S2), the bands at 1634 and 1435 cm^−1^ assigned to ν_as,s_(COO^−^) carboxylate groups can be found; these are different from the bands assigned to the acetate group (1586, 1420 cm^−1^) in the IR spectrum of the initial mixed-valence iron acetate. The strong bands at 2851, 2922, and 2954 cm^−1^ are assigned to asymmetric and symmetric stretching vibrations, ν_as,s_, of CH_3_ and CH_2_ groups, which are found in larger quantities in the structure of the fatty acid coating of the nanoparticle surface. The medium intensity bands at 3317 and 3391 cm^−1^ correspond to ν(OH) vibrations [26]. In the IR spectrum of the NPT1b sample (Supporting Information File 1, Figure S2) (the bands mentioned above) characteristic of the organic stabilizer which covers the nanoparticles, were also observed but slightly shifted (ν_as,s_(COO^−^) at 1641 and 1464 cm^−1^, while ν_as,s_(CH_3_,CH_2_) are at 2922 and 2957 cm^−1^). It is important to highlight the difference between the intensity of the band characteristic for the Fe–O bond (565 cm^−1^, very strong) and the organic material (for example, 2922 cm^−1^ assigned to CH_2_, medium) [26]. A reverse phenomenon was observed in the NPT1a spectrum, and this suggests that the content of the inorganic core in NPT1b is much higher***.*** Thermogravimetric analysis (TGA) (Supporting Information File 1, Figure S3) was used to verify this observation. Thus, by heating the samples to 700 °C, it was observed that NPT1a loses 76% of its weight, while NPT1b loses only 14%, confirming the FTIR observation. The loss of mass was ascribed to the decomposition of organic material at the surface of the nanoparticles. Therefore, the amount of inorganic core (iron oxide nanoparticles) which remained after heating was ≈24% for NPT1a and ≈86% for the NPT1b sample. The large quantity of organic stabilizer on the NPT1a nanoparticles is responsible for the better dispersion of the nanoparticles in hexane. This allowed for easier filtration and passing through the filter than for the NPT1b nanoparticles.

**Figure 1:**
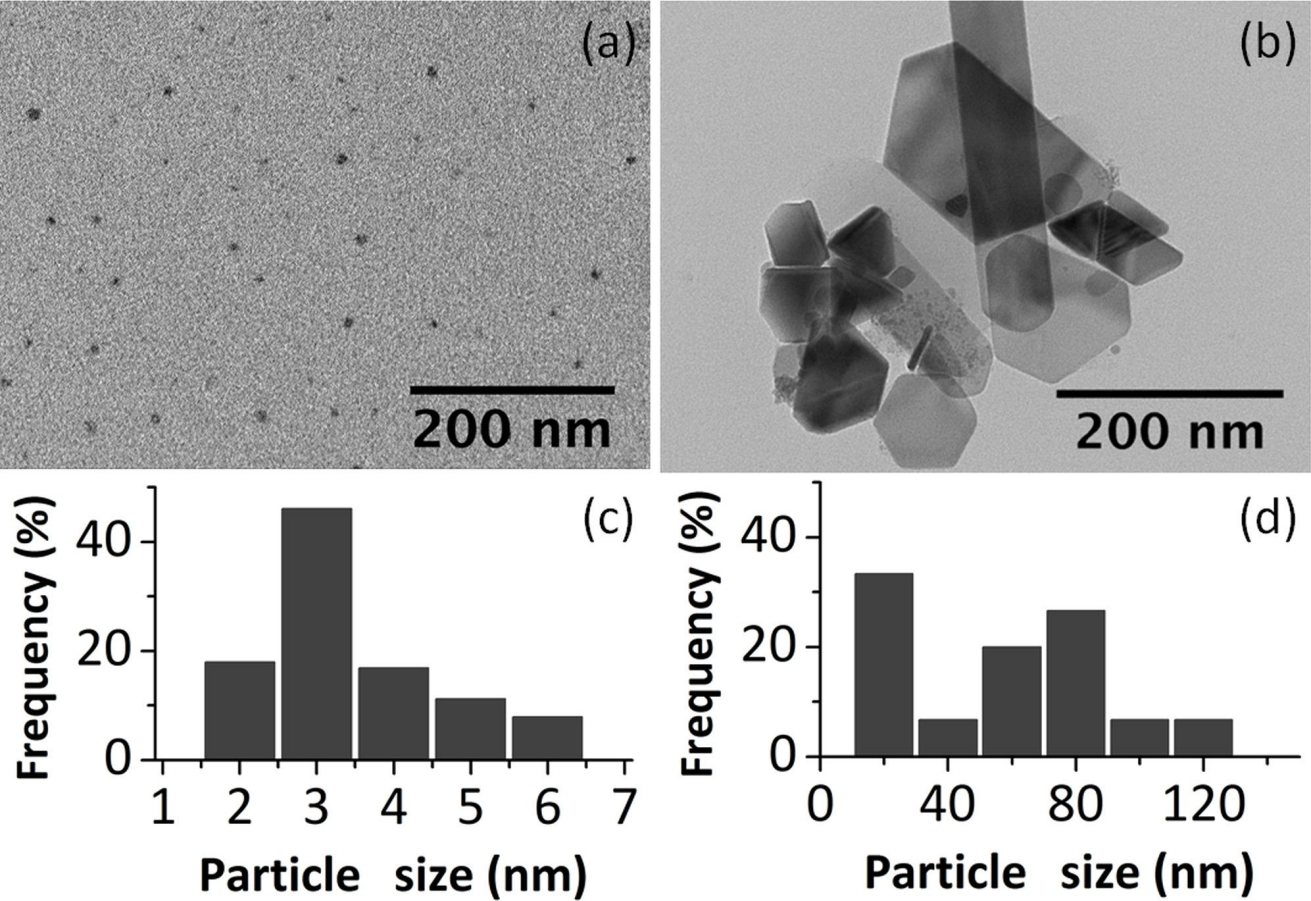
TEM images and particle size distribution histograms of NPT1a (a,c) and NPT1b (b,d).

The morphological characteristics of the samples were studied using transmission electron microscopy (TEM). Figure 1a, b shows TEM images of the two samples together with the size distribution histograms (Figure 1c,d). The geometry of NPT1a nanoparticles can be approximated as spherical with some irregularities having a diameter in the range of 2–6 nm and an average size of 3.5 nm. The morphological aspect of NPT1b nanoparticles obviously differs from that of the NPT1a sample. The NPT1b sample consists of nanoparticles of various shapes (hexagons, rods, triangles, etc.) and diameters (20–120 nm). Comparing the two samples from the same reaction batch, it is worth emphasizing that the chosen separation and purification method was extremely effective. The larger and heterogeneous NPT1b particles could be easily removed to obtain spherical small nanoparticles, NPT1a, with a narrow size distribution.

The nanoparticle dispersion and diameter distribution was evaluated by dynamic light scattering (DLS) (Supporting Information File 1, Figure S4). It was confirmed that the high amount of stabilizing agent for NPT1a provided good stability. Thereby, from the DLS measurement results, NPT1a nanoparticles have a diameter of 2.3–3.6 nm with a maximum of 2.85 nm, which is similar to the results obtained with TEM. It was not possible to measure the size of the NPT1b nanoparticles in solution because of fast sedimentation.

Wide angle X-ray diffraction (WAXD) patterns of the samples were recorded. The diffractogram of NPT1a (Supporting Information File 1, Figure S5a) does not show any peaks, demonstrating the amorphous state of this sample. The peaks found in the diffractogram of sample NPT1b (Supporting Information File 1, Figure S5b) were assigned to magnetite (JCPDS 190629).

Thus, the possibility to obtain magnetite nanoparticles from a mixed-valence iron acetate cluster has been demonstrated. In this procedure, small monodisperse nanoparticles were separated from polydisperse ones by a simple filtration.

### Preparation optimization for iron oxide nanoparticles of desired morphology

The preparation of nanoparticles with different sizes and shapes using the same equipment is of interest for industry. The optimization of the preparation process to obtain a certain (specific) morphology of nanoparticles was possible after performing different sets of experiments and varying different parameters such as temperature, reaction time and concentration of reagents and stabilizing agents. Among them, the experiments with optimized parameters that gave nanoparticle samples NPT2–NPT4 with well-defined shapes (cubic, spherical, and hedgehog-like (hairy)) were selected (Table 1). Cubic nanoparticles (NPT2) were obtained by thermal decomposition of the FeF ([Fe_3_O(C_4_H_3_OCOO)_6_(CH_3_OH)_3_]NO_3_∙2CH_3_OH) cluster in the presence of hexadecylamine (HA), olive oil (OO) and TCAA at 350 °C.

**Figure 2:**
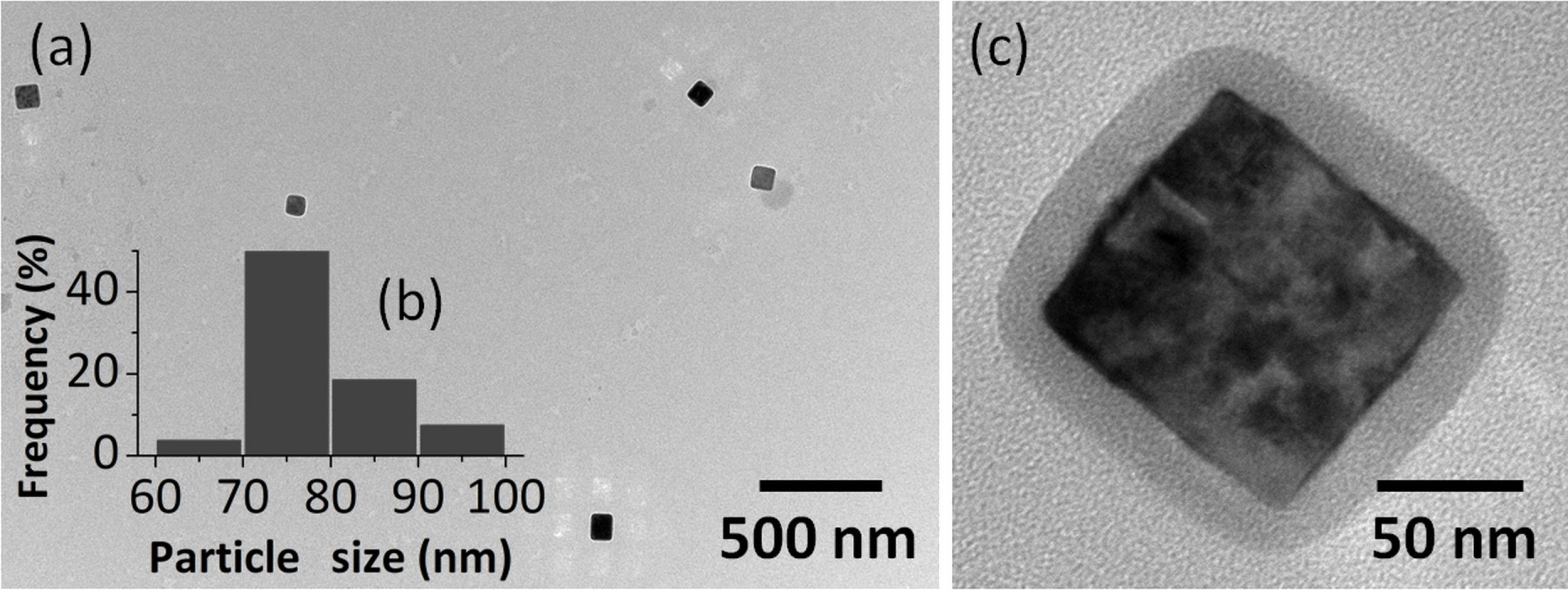
The TEM images (a,c) and the histogram of the particle size distribution (b) of NPT2 nanoparticles.

The reaction was performed at higher temperatures (350 °C) than in the case of the other samples. In Figure 2a,b the TEM images are presented and histograms of distribution by particle diameter (Figure 2c) for NPT2. The nanoparticles have an edge length of 78 ± 17 nm and 70% of the particles are ≈75 nm. At a higher magnification (Figure 2c), these particles appear as core–shell type materials, where the core has a higher contrast (indicating the presence of metal), and the shell has a lower contrast, characteristic for organic compounds (in this case, the stabilizer).

**Figure 3:**
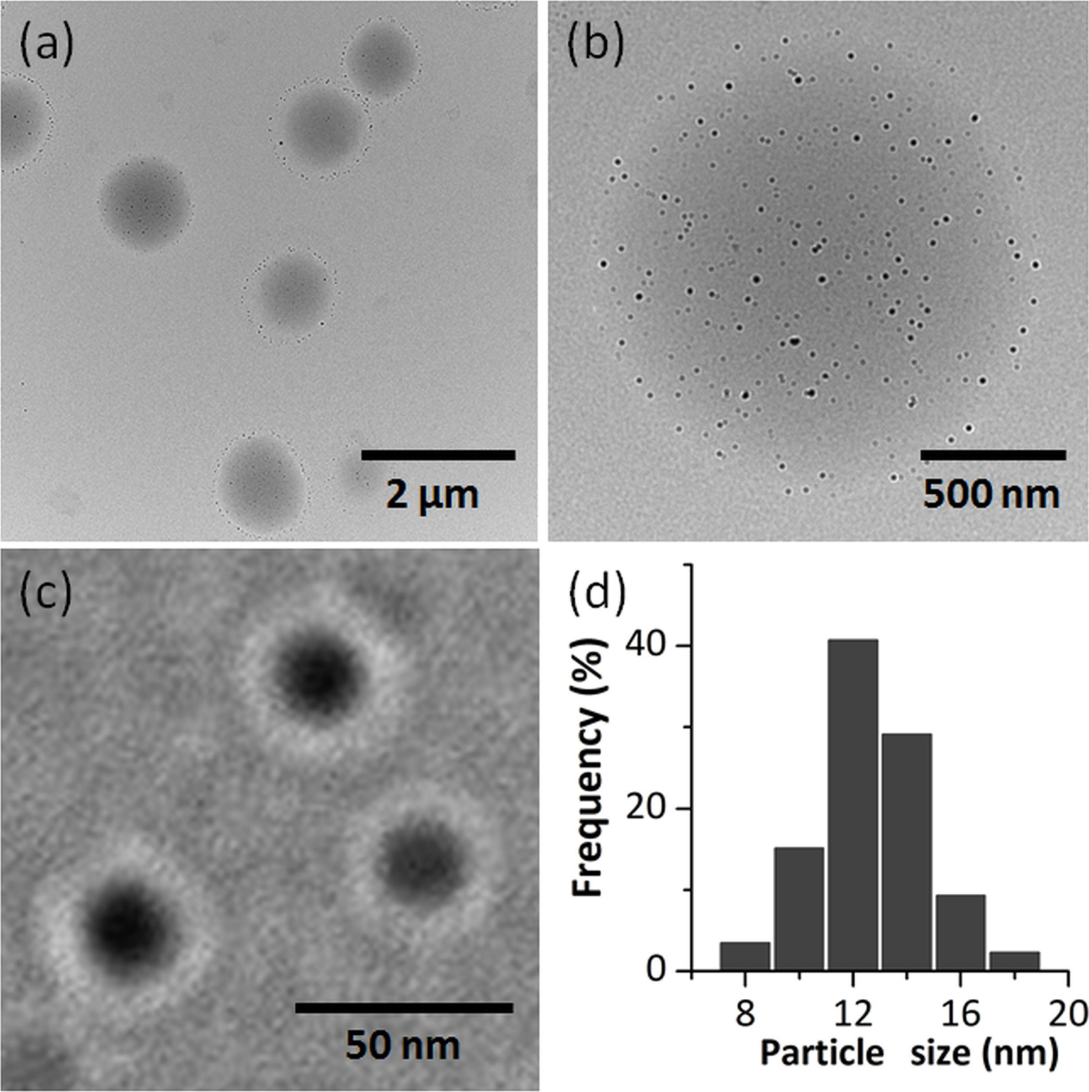
The TEM images (a–c) and the histogram of diameter distribution of NPT3 nanoparticles (d).

In Figure 3a–c the TEM images are shown and the histograms of the distribution by diameter of aggregates (Figure 3d) for sample NPT3, which was obtained from FeCrF (FeCr_2_O(C_4_H_3_OCOO)_6_(CH_3_OH)_3_]NO_3_∙2CH_3_OH). A general overview shows spheres of about 1 μm (Figure 3a), while the higher magnification images revealed that these are made of much smaller spheres (Figure 3b,c) of 12.5 ± 6 nm (Figure 3d). The core–shell morphology is visible in Figure 3c, with an inorganic core covered by the surfactant.

**Figure 4:**
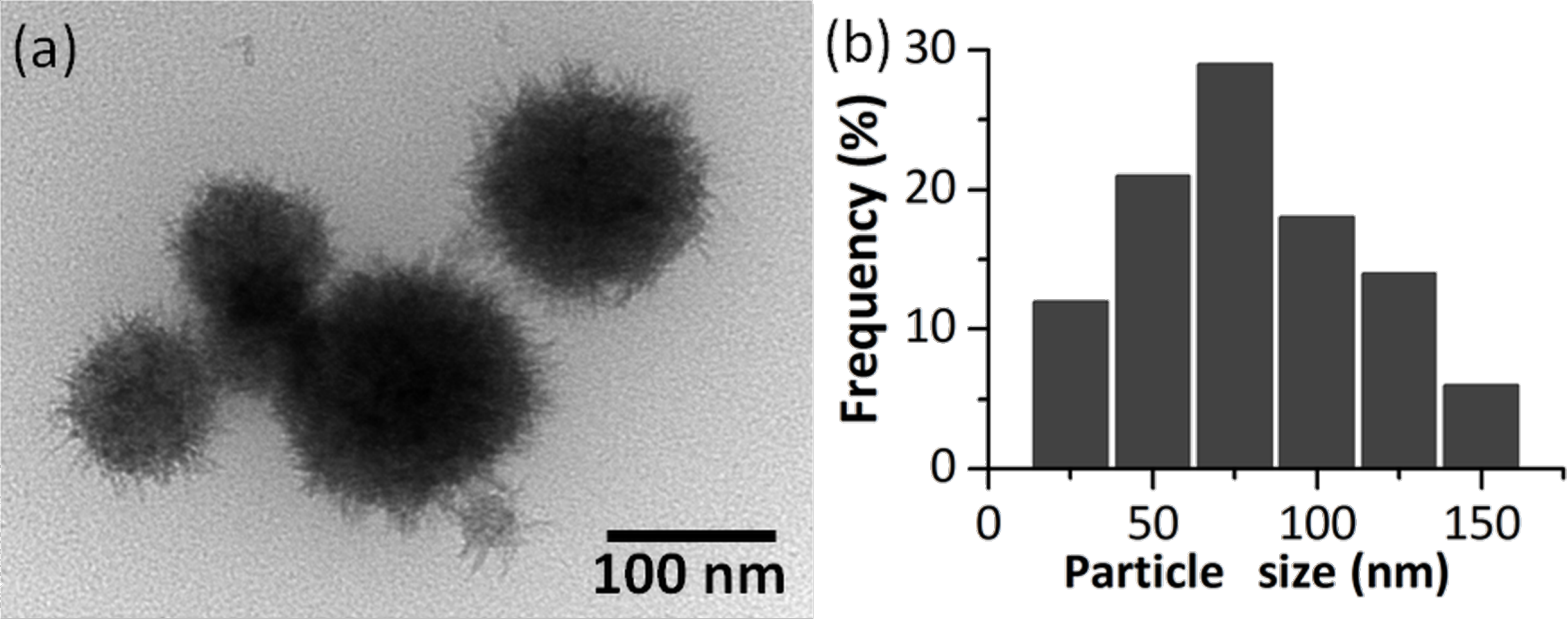
TEM image (a) and diameter distribution histogram (b) of NPT4 nanoparticles.

Nanoparticle samples (NPT4) were obtained starting from a FeAc2 ([Fe_3_O(CH_3_COO)_6_(H_2_O)_3_]NO_3_∙4H_2_O) cluster. TEM observations (Figure 4) revealed particles in the form of spheres with hair-like structures protruding from the surface at various angles, but generally in a radial direction from the sphere center. The diameter of the spheres is 80 ± 60 nm, and the hairs are of 20 ± 5 nm length and 2 ± 0.5 nm width.

The analysis of the samples by IR spectroscopy (Supporting Information File 1, Figure S6) revealed the presence of stabilizing agents on the surface of the particles. As discussed above, the bands at 2853–2955 cm^−1^ attributed to symmetric and asymmetric stretching vibrations of the saturated C–H bond of the groups CH_2_ and CH_3_ [27] are present in the spectra of all samples, indicating the presence of long chain stabilizing agents on the surface of the nanoparticles. The bands at 382–619 cm^−1^ are characteristic of the metal oxides [26]. A particular case is sample NPT3, containing iron/chromium mixed oxides, which are present in the IR spectrum. This is in addition to the bands characteristic for Fe–O bond vibrations, the band at 555 cm^−1^, which may be attributed to the Cr–O lattice vibration [28]. The presence of metals in the samples was confirmed by SEM-EDX spectroscopy. In the spectra of the samples NPT2 and NPT4, the iron was detected, while in NPT3 (Supporting Information File 1, Figure S7), both iron and chromium were present with an atomic ratio (Cr/Fe) of 1.83, similar to the starting cluster FeCrF (Cr/Fe 1.88). The thermogravimetric data (Supporting Information File 1, Figure S8) shows a residual mass of 69% for NPT2 at 600 °C, 63% for NPT3 and 22% for NPT4. The loss of mass starts around 50−90 °C due to the evaporation of solvent from the surface of the nanoparticles. After this first step, there are three consecutive stages of decomposition that start around 175, 335 and 400 °C, and correspond to the evaporation/decomposition of organic material at the surface of nanoparticles. The amount of organic material lost at high temperature for each sample demonstrates that sample NPT4 contained a larger amount of stabilizers compared with samples NPT2 and NPT3.

The samples NPT2–NPT4 were analyzed by powder X-ray diffraction. The diffractograms of samples NPT3 and NPT4 do not present any peaks, indicating that both samples are amorphous, while the diffractogram of sample NPT2 (Supporting Information File 1, Figure S9) shows diffraction peaks, which coincide with those from the JCPDS 04-0755 database and are characteristic for maghemite (γ-Fe_2_O_3_).

The morphology of the nanoparticles was studied with TEM, which evidenced the differences between samples depending on the precursor used and reaction conditions. Thus, we find that by this method, it is possible to control the size and shape of the nanoparticles by adjusting the concentration and type of precursors, reaction temperature and time (Table 1). We presented only the particles with well-defined morphology, while in other cases, morphological polydispersity was observed. Here just several examples were shown. By varying reaction parameters, it is possible to obtain nanoparticles with more sizes and shapes.

### Solvothermal method

Samples NPS1–NPS3 were prepared by a solvothermal method (Supporting Information File 1, Figure S10) from a mixture of μ_3_-oxo trinuclear iron acetate ([Fe_3_O(CH_3_COO)_6_(H_2_O)_3_]NO_3_·4H_2_O), FeAc2, used as the source of iron, sodium oleate (NaOI) and/or DA used as stabilizing agents, and TCAA as solvent. Three reactions were carried out simultaneously, and for each of them the same amounts of FeAc2, TCAA and NaOI were used, but with different additions of DA (Table 2).

**Table 2:** The experimental conditions used for conversion of the iron-based precursor FeAc2 into nanoparticles with different shapes and sizes by solvothermal approach (reaction time: 30 min).

Sample	Feed reactants				Nanoparticle characteristics	
	Precursor	Surfactant	Solvent	*T* (°C)	Diameter (nm)	Morphology

NPS1	FeAc2 (0.7 mmol)	NaOl (0.8 mmol), DA (5.4 mmol)	TCAA (6.1 mmol)	250	10	Irregularly shaped nanoparticles
NPS2	FeAc2 (0.7 mmol)	NaOl (0.8 mmol), DA (1.6 mmol)	TCAA (6.1 mmol)	250	7 (1.5 × 20)^a^	Nanowires and spherical nanoparticles
NPS3	FeAc2 (0.7 mmol)	NaOl (0.8 mmol)	TCAA (6.1 mmol)	250	55	Spherical
NPS4	FeAc2 (0.4 mmol)	NaOl (0.7 mmol), DA (8 mmol)	TCAA (12.2 mmol)	250	5	Irregular shapes
NPS5	FeAc2 (0.4 mmol)	NaOl (0.7 mmol), DA (8 mmol)	IPA (12.2 mmol)	250	6	Irregular shapes
NPS6	FeAc2 (0.4 mmol)	NaOl (0.7 mmol), DA (8 mmol)	DMF (12.2 mmol)	250	6	Irregular shapes
NPS7	FeAc2 (1.4 mmol)	DA (5.4 mmol)	TCAA (9.2 mmol)	200	5	Spherical with some irregularities
NPS8	FeAc2 (1.4 mmol)	NaOl (0.8 mmol), DA (5.4 mmol)	TCAA (9.2 mmol)	200	3	Spherical with some irregularities
NPS9	FeAc2 (1.4 mmol)	NaOl (3.2 mmol), DA (5.4 mmol)	TCAA (9.2 mmol)	200	74	Various shapes (hexagonal, cubic, etc.)

^a^Width × Length of nanowires.

Analyzing the IR spectra of the samples NPS1–NPS3 (Supporting Information File 1, Figure S11a), characteristic bands were observed for carboxyl groups at 1649–1641 cm^−1^ and 1439 cm^−1^. The bands at 2853–2851 cm^−1^ and 2922 cm^−1^ are attributed to CH_2_ groups, which are characteristic of fatty acids [26]. Absorption bands characteristic for CH_3_ groups were identified at 2955 cm^−1^ in the spectra of all the samples. From the data obtained by IR spectroscopy it can be deduced that nanoparticle samples NPS1–NPS3 are coated with organic material (stabilizing agents). The presence of iron in the samples NPS1–NPS3 was confirmed by XRF spectroscopy (Supporting Information File 1, Figure S11b).

The residual mass at the end of the temperature range investigated by TGA (700 °C) was 62.61% for NPS1, 63.37% for NPS2 and 30.36% for NPS3, respectively (Supporting Information File 1, Figure S12). From the TGA data, it can be seen that with a decreasing amount of DA used in the process of obtaining nanoparticle samples NPS1–NPS3, the percentage of decomposed material also decreases, which most likely consists of the stabilizers from the surface of the particles. From the WAXD patterns (Supporting Information File 1, Figure S13) it was determined that NPS2 and NPS3 samples are amorphous, while the diffraction peaks present in the diffractogram of the NPS1 sample was assigned to α-Fe_2_O_3_ (JCPDS 300664).

**Figure 5:**
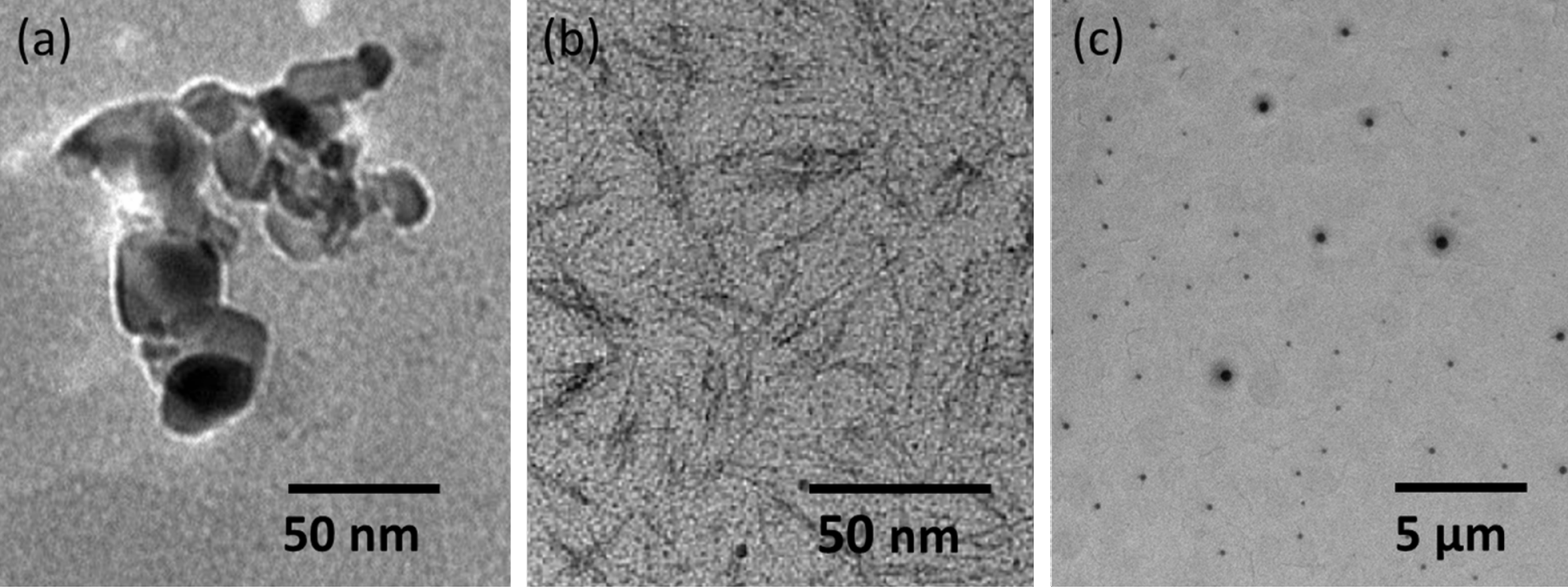
TEM images of samples NPS1 (a) NPS2 (b) and NPS3 (c).

TEM images (Figure 5) and size distribution histograms (Supporting Information File 1, Figure S14) provide information on the shape and size of particles analyzed. The NPS1 sample (Figure 5a) shows irregularly shaped nanoparticles with diameters of 7–44 nm, and an average diameter of 10 nm. For sample NPS2 (Figure 5b), both spherical nanoparticles with an average size of 7 nm, and nanowires of 20 nm length and 1.5 nm width were obtained. Nanowires constitute the majority of the particles in the NPS2 sample. Nanoparticles in the NPS3 sample (Figure 5c) were of spherical shape with a diameter of 20–146 nm, and an average size of 55 nm. Thus, the DA concentration influences both the morphology and shape of the nanoparticles obtained (Table 2).

**Figure 6:**
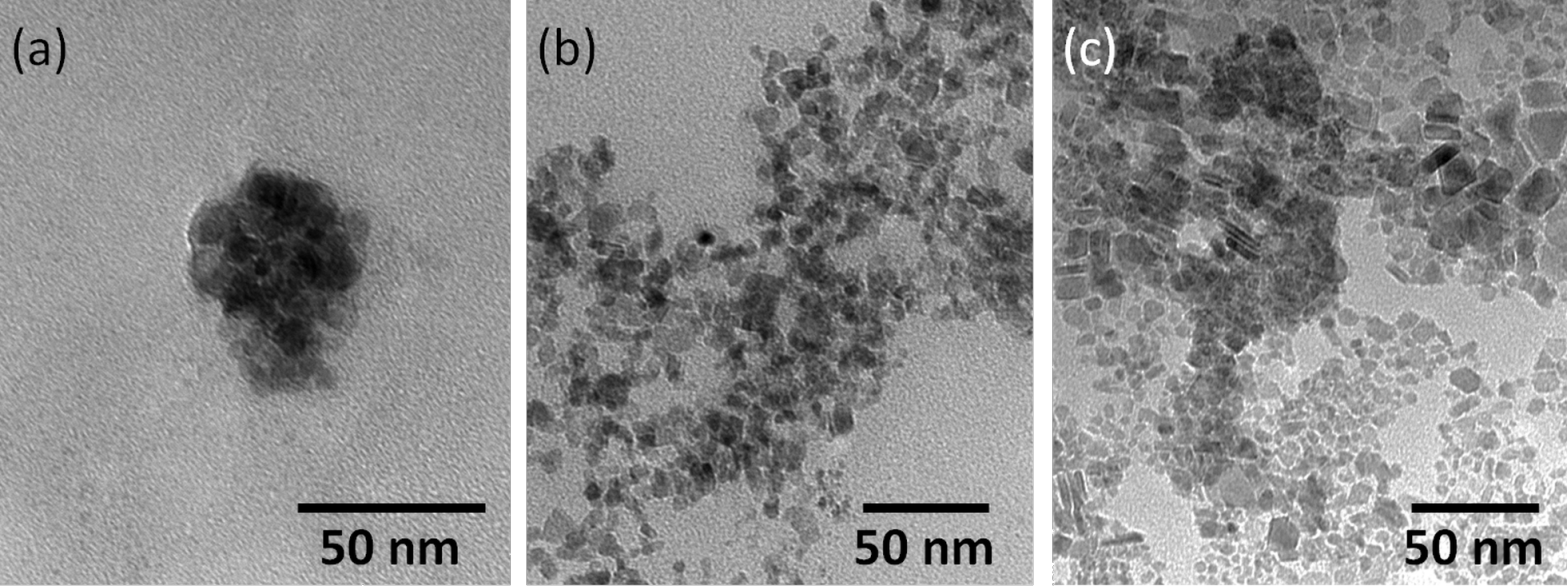
TEM images for NPS4 (a) NPS5 (b) and NPS6 (c).

By the same approach, the influence of other reagents (and their concentrations) on the morphology and size of the obtained iron oxide nanoparticles has been studied. For example, three reactions were carried out concomitantly, starting from the same concentration of FeAc2, NaOl and DA, but using different solvents. For the preparation of NPS4–NPS6, TCAA, isopropyl alcohol (IPA) and dimethylformamide (DMF) were used, respectively. It can be seen in TEM images (Figure 6) that the morphology of the nanoparticles obtained is not much different from sample to sample, and that they have irregular shapes. The sizes of obtained nanoparticles are: 3–8 nm for sample NPS4, with an average of 5 nm; 3–10 nm for sample NPS5, with an average of 6 nm and 2–16 nm for sample NPS6, with an average of 6 nm (Supporting Information File 1, Figure S15).

**Figure 7:**
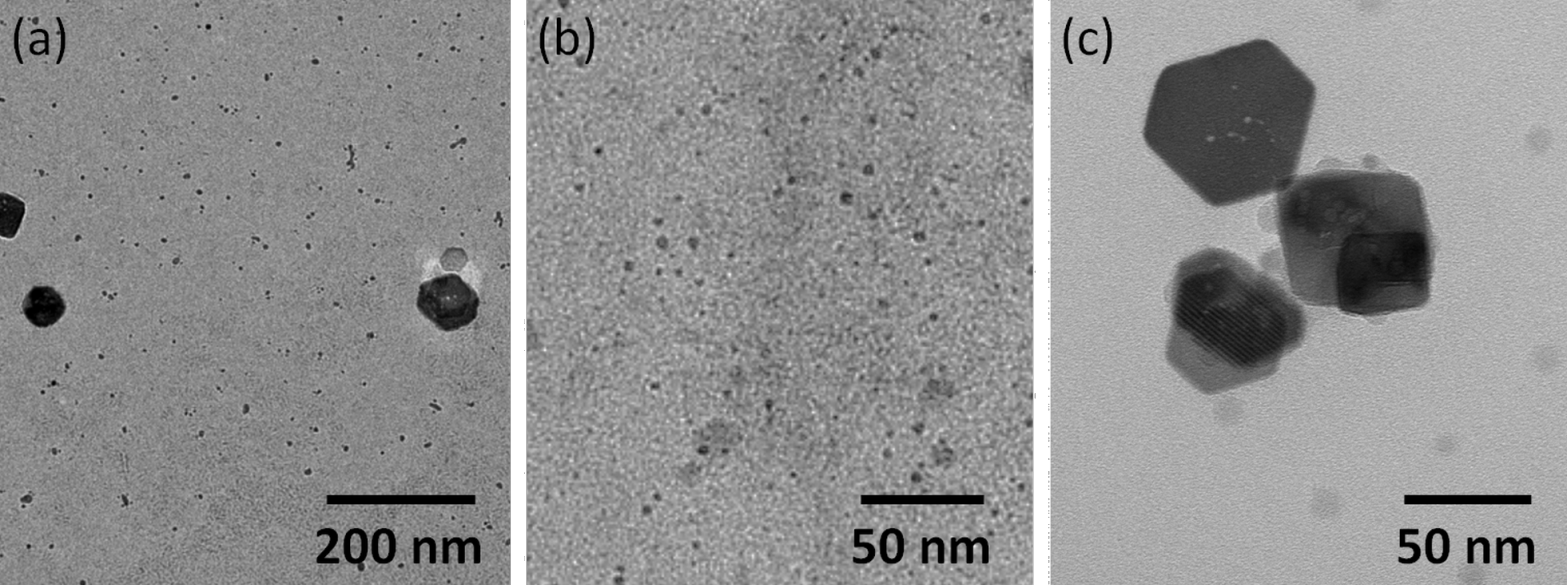
TEM images for NPS7 (a) NPS8 (b) and NPS9 (c).

By performing three simultaneous reactions, where the same concentration of FeAc2, DA and TCAA were used, it was possible to study the influence of the NaOI concentration on the shape and size of the nanoparticles. TEM images of samples NPS7–NPS9 are shown in Figure 7, and the corresponding size distribution histogram in Supporting Information File 1, Figure S16. In the case where no NaOI was used (NPS7), nanoparticles in the diameter range of 2–60 nm with an average diameter of 5 nm were obtained. Most of the nanoparticles have a diameter of up to 10 nm (Figure 7a). When NaOI was used in small amount (NPS8), nanoparticles with average diameter of 3 nm (Figure 7b) were obtained, while increasing the concentration of NaOI (NPS9) resulted in polydispersed particles of about 74 nm diameter (Figure 7c). Therefore, it is concluded that the NaOI concentration influences the shape and size of resulting nanoparticles. However, it seems that there is an optimum concentration (Table 2) that leads to nanoparticles of minimum polydispersity.

Performing several simultaneous reactions by the solvothermal method, it was demonstrated that the size and shape of the obtained nanoparticles is highly sensitive to the concentration of surfactants. By changing the solvent, the reaction outcome was not significantly affected.

### Calcination

The simplest method used to obtain nanoparticles from iron clusters is dry thermal decomposition (calcination). Different precursors were prepared and used to this end: the trinuclear iron acetate cluster (FeAc2), trinuclear iron/chromium furoate cluster (FeCrF) and an iron complex based on a macromolecular ligand containing a siloxane moiety (polyazomethine FePAZ [29]). These were calcined in an oven at 600 °C for 5 h in air heating at a rate of 50 °C/min (Supporting Information File 1, Figure S17). The obtained residues of the three complexes were collected, labelled as NPC1, NPC2, and NPC3, respectively, and analyzed further.

The bands at 548, 473 (467) and 384 cm^−1^ in the IR spectra of NPC1 and NPC3 are typical for Fe–O from hematite (α-Fe_2_O_3_) [30] (Supporting Information File 1, Figure S18). The additional band at 584 cm^−1^ in the IR spectrum of NPC2, which was not found in samples NPC1 and NPC3, was assigned to the Cr–O bond. The iron oxide nanoparticles can absorb different ions at their surface or can store different compounds in their pores. Therefore, the bands observed in the IR spectra of NPC1 and NPC2 in the region 3435– 3119 cm^−1^ and at 1400 cm^−1^ were assigned to the –OH bond [26], probably from adsorbed moisture, while the bands in the region 1700–900 cm^−1^ were assigned to the C–O bond [31] of residual organic moieties from the precursors after calcination. The presence of NPC3 in the spectra corresponding to the bands characteristic for the Si–O bond (1094 and 804 cm^−1^) and the absence of the bands characteristic for –OH and CO bonds supports the idea that sample NPC3 is an iron oxide–silica hybrid. The EDX spectrum (Supporting Information File 1, Figure S19) indeed confirms the presence of both Si and Fe in a 2.1:1 atomic ratio in the sample NPC3. In the EDX spectrum of sample NPC1, only iron was detected, while in NPC2 we could observe the peaks characteristic for Fe and Cr (Supporting Information File 1, Figure S20). An atomic ratio of ≈2 between Cr and Fe was estimated, similarly as for the starting cluster FeCrF.

**Figure 8:**
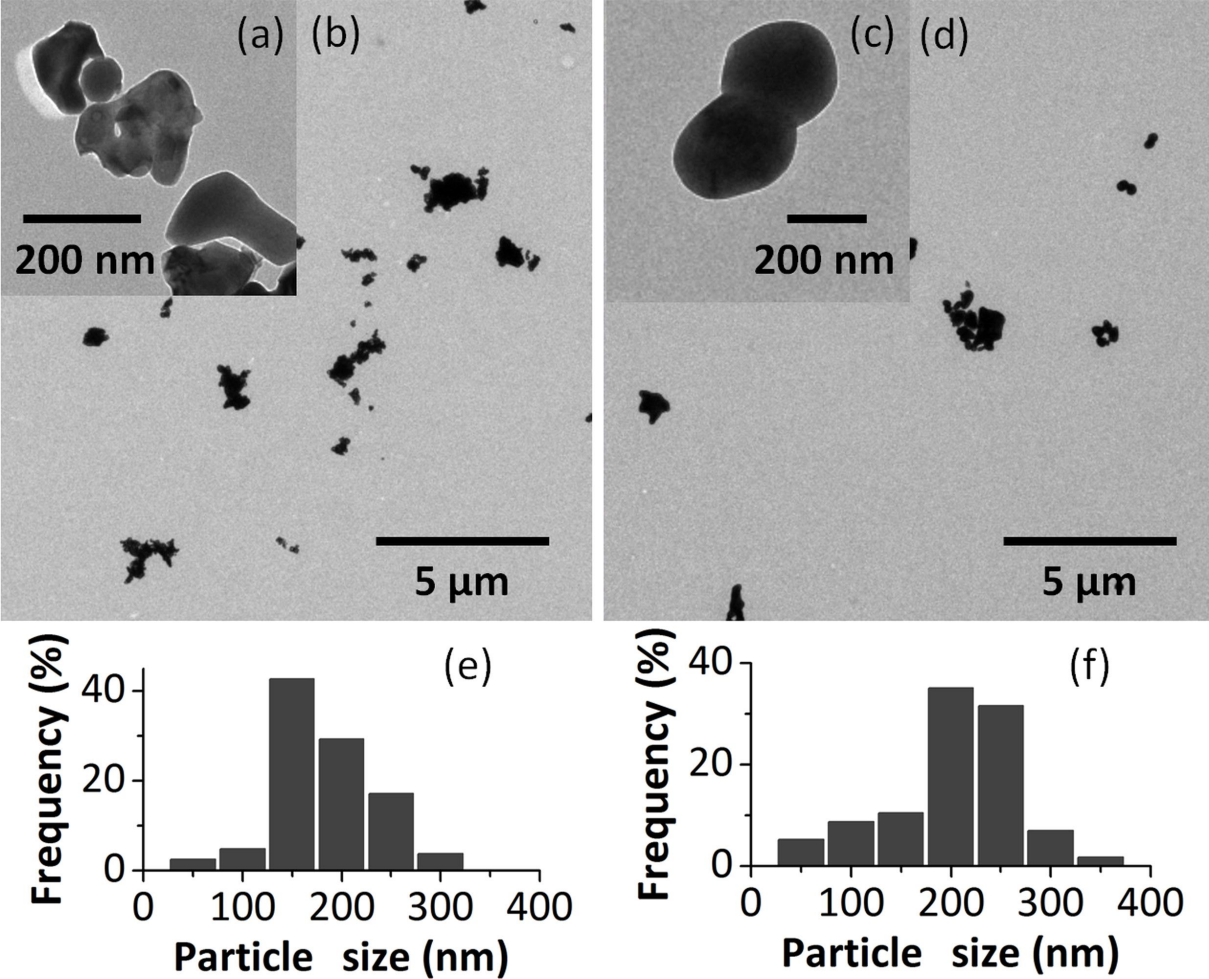
TEM images (top, a–d) and size distribution histograms (bottom, e,f) of NPC1 (a,b,e) and NPC2 (c,d,g) samples.

The TEM images and size distribution histograms of samples NPC1 and NPC2 are presented in Figure 8. In both cases, it could be observed that the obtained particles have a diameter in the range of 50–350 nm with an average diameter of 183 nm for the NPC1 sample and 203 nm for NPC2.

**Figure 9:**
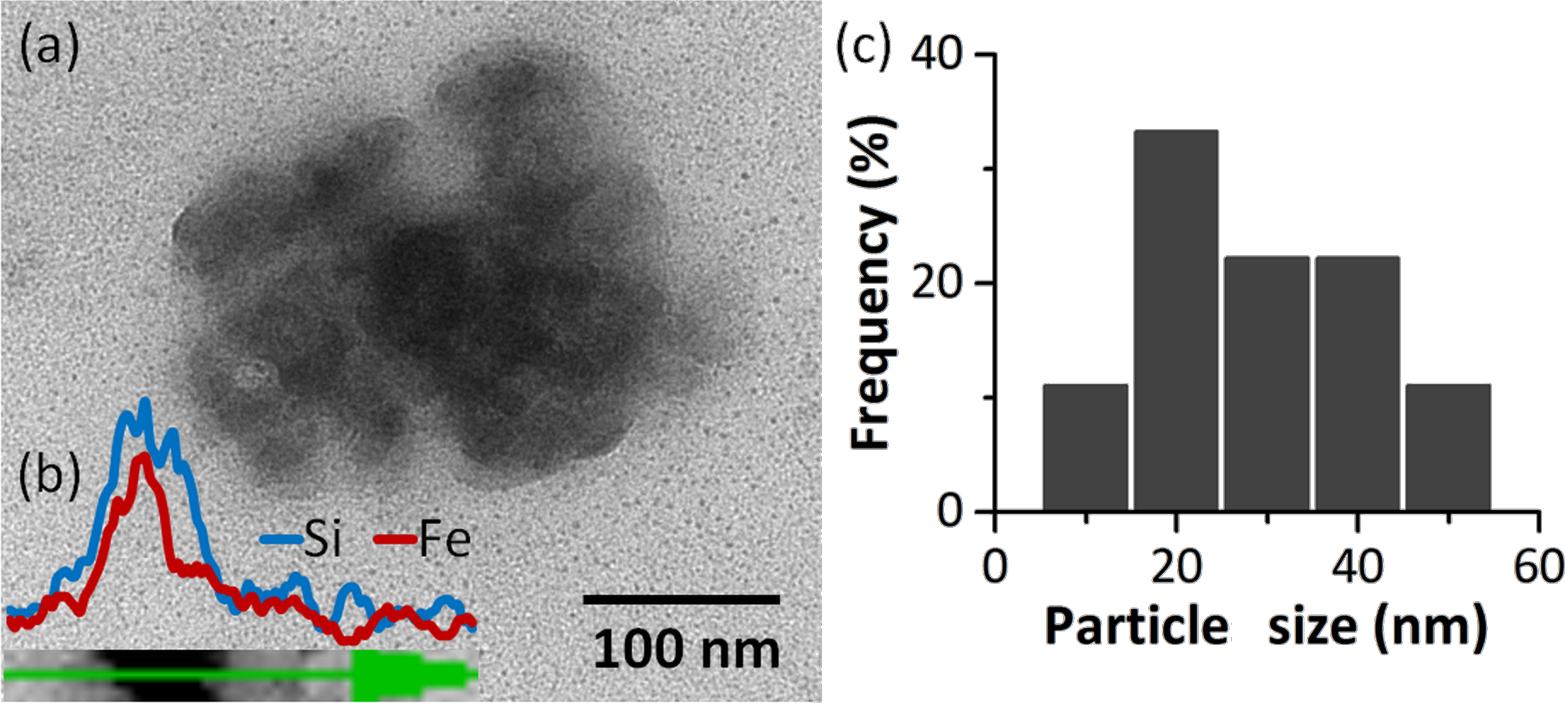
TEM image (a), EDX line scan (b) and size distribution histogram (c) of the NPC3 sample.

A completely different morphology can be seen in the case of the NPC3 sample. The TEM images (Figure 9a) show the formation of particles of irregular shape covered with a lower contrast material which might be silica. The diameter of the particles is in the range of 10–50 nm and the average size is 29 nm. EDX analysis (along the direction indicated in the TEM (Figure 9b)) made on aggregated nanoparticles showed both Fe and Si, suggesting that the iron oxide nanoparticles are covered with silica. The formation of a silica shell on the surface of the iron oxide nanoparticles in NPC3 might be the reason for such a difference of size between nanoparticles obtained in a similar way from the cluster with Si (29 nm) and without Si (≈200 nm).

Comparing the diffraction peaks identified in the WAXD patterns of NPC1–NPC3 samples (Supporting Information File 1, Figure S21) with those from the ICDD database for different oxides, it was determined that the peaks in the NPC1 and NPC3 spectra coincide with those of the well-established structure of hematite (JCPDS 33-0664), while the peaks found in the NPC2 diffractogram coincide with those of Cr_1.3_Fe_0.7_O_3_ (JCPDS 35-1112). Thus, the XRD results are in good agreement with IR and EDX observations in terms of oxide structure.

### Microwave irradiation

Iron oxide nanoparticles (NPM series) were obtained by a procedure which consists of the decomposition of iron trinuclear μ_3_-oxo acetate ([Fe_3_O(CH_3_COO)_6_(H_2_O)_3_]NO_3_·4H_2_O), FeAc2, in a strongly alkaline medium by microwave irradiation (Supporting Information File 1, Figure S22). In order to obtain NPM2 and NPM0 oxides, the pH was adjusted to 12, while for NPM1 the pH of the reaction system was adjusted to 11. The sample NPM0 was obtained by adding ammonia water to the aqueous solution of FeAc2, without any additional operations (stirring, heating, etc.) and used as a control. The NPM1 and NPM2 samples were obtained by adding ammonia water to an aqueous solution of FeAc2 and irradiated with a microwave irradiation (300 W) at 70 °C for 5 min. The precipitate obtained in all three cases was washed with distilled water to adjust the pH to a middle value.

The IR spectra of NPM0–NPM2 samples are similar (Supporting Information File 1, Figure S23a). The vibration bands characteristic for Fe–O bonds were identified in the 620–450 cm^−1^ range, while the bands in the region 3435–3119 cm^−1^ and 1400 cm^−1^ are attributed to the –OH bond vibration [32]. The presence of iron in the samples NPM0–NPM2 was confirmed by EDX analysis (Supporting Information File 1, Figure S23b).

**Figure 10:**
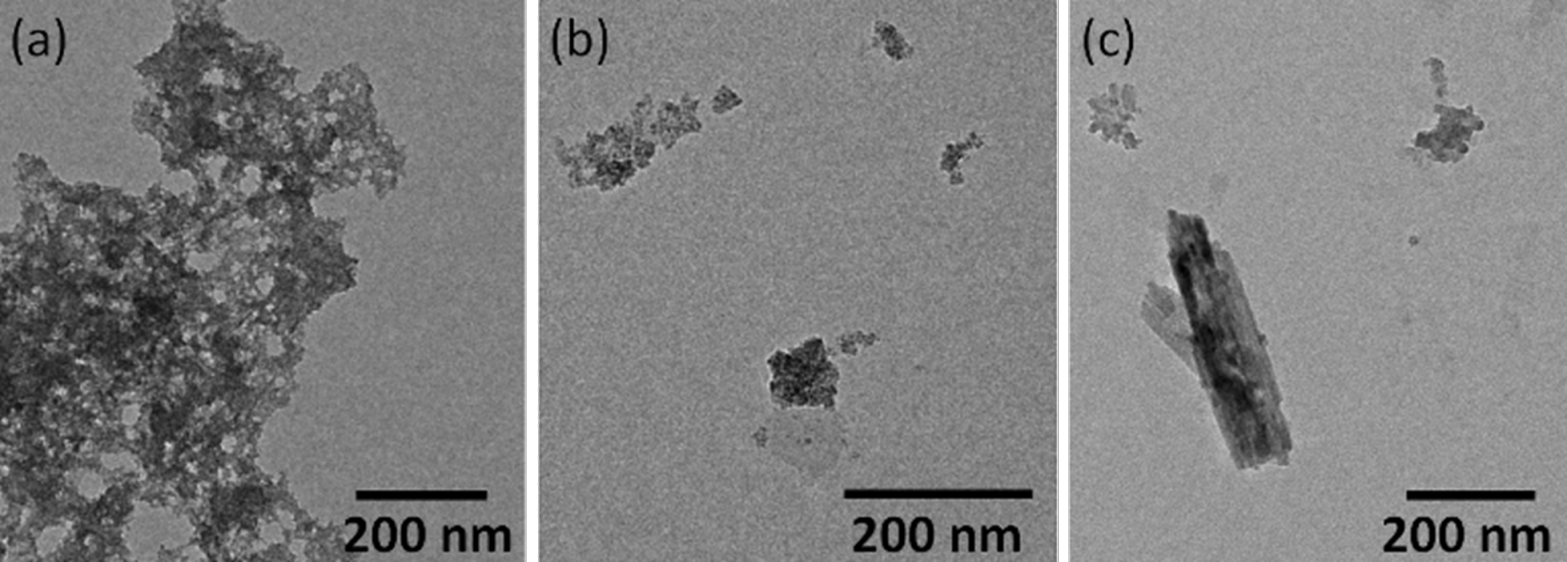
TEM images of NPM0 (a), NPM1 (b) and NPM2 (c).

TEM images of NPM0–NPM2 samples are shown in Figure 10. Samples NPM0 and NPM1 consist of irregularly shaped particles. In the NPM2 sample, prepared at a different temperature as compared with NPM0 and different pH compared with NPM1 sample, a change in morphology is observed due to changes in pH. Here, in addition to the irregularly shaped particles with a diameter of about 10 nm, agglomerates of 80–100 nm in width were observed, which in turn consist of rods approximately of 15 nm in width.

From the WAXD diffractograms (Mo anode) of the NPM0–NPM2 samples (Supporting Information File 1, Figure S24a) it was observed that NPM0 and NPM1 samples are amorphous, while the NPM2 sample is crystalline. The peaks identified in this case are typical for α-Fe_2_O_3_ (JCPDS 33-0664). Raman spectra were also recorded (Supporting Information File 1, Figure S24b) to confirm the oxide phase. The bands detected in the spectra at 223, 290, 406, 609 and 659 cm^−1^ are similar for all samples NPM0–NPM2 and were assigned to α-Fe_2_O_3_ (hematite) [30].

### Ultrasonication

Iron oxide nanoparticles (NPU1–NPU2) were obtained using a similar reaction mixture as for preparation of the NPM series but subjected to irradiation with ultrasound (Supporting Information File 1, Figure S22). The procedures for samples NPU1–NPU2 differ only in the ultrasonic irradiation time (5 min for NPU1 and 30 min for NPU2). In addition, sample NPU2 was also subsequently subjected to thermal treatment at 400 °C to track changes in morphology and crystallinity (NPU2T). The IR absorption bands characteristic of iron oxide (Fe–O bond) are present in the 617–407 cm^−1^ spectral range (Supporting Information File 1, Figure S25a). The presence of Fe in the samples NPU1–NPU2 was confirmed by EDX analysis (Supporting Information File 1, Figure S25b).

**Figure 11:**
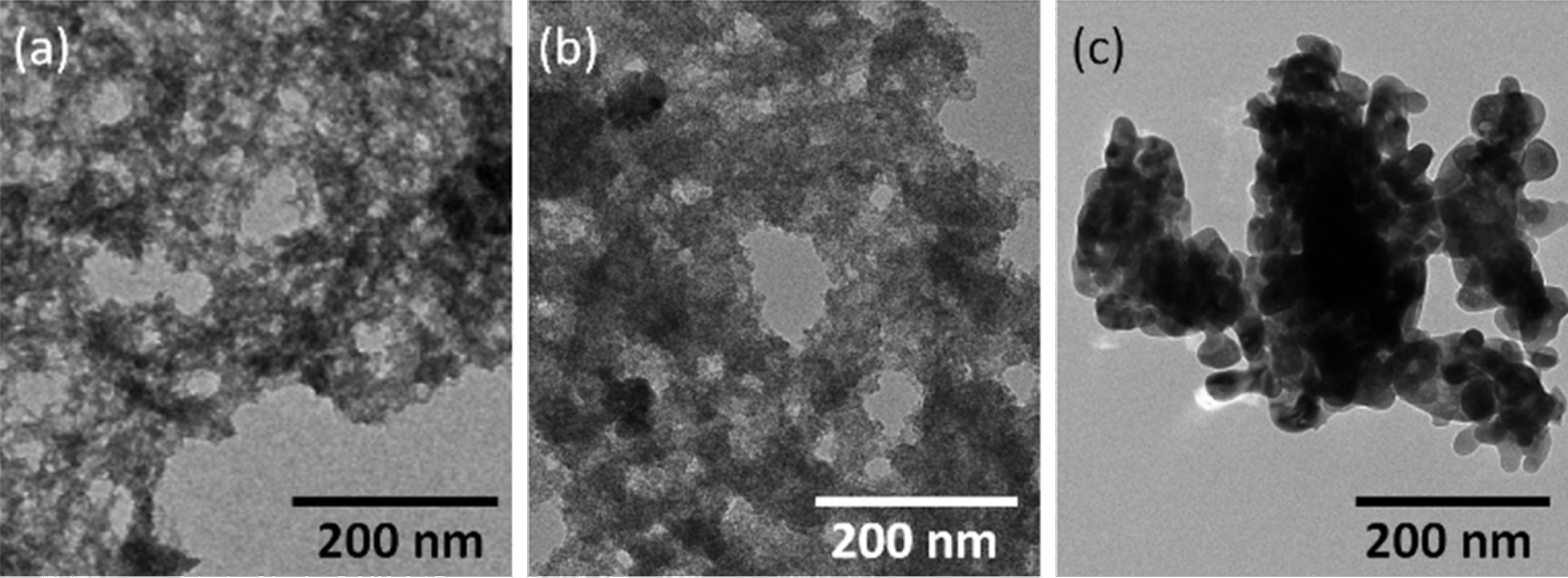
TEM images of NPU1 (a), NPU2 (b) and NPU2T (c).

In Figure 11, TEM images of the samples NPU1 (a), NPU2 (b), and NPU2T (c) are shown. Samples NPU1 and NPU2 appear as irregularly shaped materials. After thermal treatment of the sample NPU2, the formation of spheroidal aggregates of nanoparticles with a diameter of ≈20 nm can be seen.

WAXD diffractograms (Mo anode) for samples NPU1, NPU2 and NPU2T are shown in Supporting Information File 1, Figure S26. Diffractograms for samples NPU1 and NPU2 contain no diffraction peak, which indicates the amorphous nature of the samples, while the diffractogram of sample NPU2T contains diffraction peaks typical for hematite (JCPDS 33-0664).

## Conclusion

Using homonuclear and heteronuclear iron clusters as precursors, iron oxide or mixed oxide nanoparticles were obtained through various approaches: thermal decomposition, solvothermal method, calcination, microwave irradiation and ultrasonication. Taking into consideration the scientific and industrial interest in controlling the morphology of nanoparticles, the influence of reaction parameters on the final products was studied. With the use of thermal decomposition, we show the possibility to obtain nanoparticles of desired shape (cubic, spherical, hedgehog-like) by changing the type and amount of surfactants, reaction temperature and time. By thermal decomposition followed by simple filtration, monodisperse nanoparticles were obtained and separated from polydisperse nanoparticles. Starting from the similar reaction mixture as in the thermal decomposition procedure, by using solvothermal method, it was demonstrated that the morphology of the final nanoparticles is very sensitive to the amount of stabilizing agents (DA and NaOI) and there is an optimal concentration at which we could obtain nanoparticles with controlled morphology. By the simple calcination procedure, it was found that using iron or iron/chromium carboxylate clusters, polydisperse nanoparticles of ≈200 nm are obtained, while using an iron precursor with silicone ligand, iron oxide–silica hybrid nanoparticles of just ≈30 nm in diameter can be prepared. It has been shown that the microwave irradiation and ultrasonication times of μ_3_-oxo trinuclear iron(III) acetate influences both the crystallinity and shape of nanoparticles. As a result of this apparently wide and heterogeneous investigation, we are able to design and prepare nanoparticles with well-defined shape, size, morphology and composition, according to specific requirements. After testing a significant number of methods with the same precursors and varying reaction parameters, we consider that the most reliable method for the preparation of iron oxide NPs with well-defined size and shape would be the thermal decomposition method, while other methods also allow for tuning of the nanoparticle diameter (within the 2–350 nm range).

## Experimental

### Materials

The mixed-valence iron trinuclear acetate [Fe_2_^III^Fe^II^O(CH_3_COO)_6_(H_2_O)_3_]∙2H_2_O, denoted by FeAc1, was prepared by a procedure reported in the literature [33] using FeCl_3_∙6H_2_O and FeCl_2_·4H_2_O at a molar ratio of 1:2, calcium acetate ((CH_3_COO)_2_Ca) and glacial acetic acid. The identity of the compound obtained was confirmed by FTIR (KBr) *ν*: 3421 (s), 1586 (vs), 1420 (vs), 1349 (m), 1050 (w), 1033 (w), 715 (m), 663 (s), 618 (m), 468 (w), 561 (w); Anal. calcd for C_12_H_28_O_18_Fe_3_: C, 23.00; H, 4.49; found: C, 23.22; H, 4.57; Mossbauer spectroscopy measurements (Doublet 1: δFe 1.27; ΔEQ 2.68; Γ 0.56; Doublet 2: δFe 0.56; ΔEQ 0.81; Γ 0.54).

The compound FeAc2, μ3-oxo trinuclear iron(III) acetate ([Fe_3_O(CH_3_COO)_6_(H_2_O)_3_]NO_3_∙4H_2_O), was prepared by a previously reported procedure [34] by using Fe(NO_3_)_3_∙9H_2_O and CH_3_COONa, glacial acetic acid and distilled water. The structure of the compound was confirmed by FTIR and single crystal XRD (lattice parameters). FTIR (KBr) *ν*: 420 (vw), 468 (vw), 527 (w), 613 (s), 662 (s), 822 (w), 835 (w), 898 (vw), 951 (w), 1035 (m), 1292 (s), 1385 (vs), 1450 (vs), 1589 (vs), 1688 (s), 2545 (vw), 2636 (vw), 3413 (s).

Iron furoate [Fe_3_O(C_4_H_3_OCOO)_6_(CH_3_OH)_3_]NO_3_∙2CH_3_OH (FeF) was obtained as reddish single crystals following the procedure described by Melnic et. al. [35] from a mixture of Cu_2_(C_4_H_3_OCOO)_4_∙4H_2_O, Fe(NO_3_)_3_∙9H_2_O and methanol. The structure of the compound was confirmed by FTIR and single crystal XRD (lattice parameters). FTIR (KBr) *ν*: 3435 (s), 3154 (s), 1591 (vs), 1564 (s), 1510 (vs), 1437 (vs), 1385 (vs), 1369 (vs), 1219 (s), 1155 (s), 1080 (m), 1007 (m), 972 (s), 874 (s), 845 (w), 826 (w), 781 (s), 743 (m), 638 (s), 602 (s), 515 (s). Anal. calcd for C_35_H_38_NO_27_Fe_3_: C, 39.20; H, 3.57; N, 1.31; found C, 39.72; H, 3.16; N, 1.54.

The heteronuclear iron/chromium furoate, FeCr_2_O(C_4_H_3_OCOO)_6_(CH_3_OH)_3_]NO_3_∙2CH_3_OH (FeCrF), was prepared by the solvothermal method using a mixture of Cu_2_(C_4_H_3_OCOO)_4_·4H_2_O (0.42 g), Fe(NO_3_)_3_∙9H_2_O (0.25 g), and Cr(NO_3_)_3_∙9H_2_O (0.5 g) in 5 mL methanol. The process took place at 60 °C for 5 h, with heating and cooling rates of 0.1 °C/min. Dark brown reddish single crystals were observed directly in the autoclave at the end of the process. The structure of the compound was confirmed by FTIR, EDX and single crystal XRD (lattice parameters). FTIR (KBr) *ν*: 3618 (vw), 3140 (vw), 1605 (s), 1582 (s), 1475 (vs), 1423 (vs), 1377 (s), 1232 (m), 1205 (s), 1142 (m), 1078 (w), 1016 (m), 937 (m), 885 (w), 802 (w), 793 (vw), 779 (s), 640 (w), 615 (w), 594 (w), 519 (w), 376 (vw); EDX (Fe/Cr 1:1.9).

FePAZ ({[N(CH_2_)_3_Si(CH_3_)_2_]_2_O[CHC_6_H_4_OCH_2_Si(CH_3_)_2_]_2_O∙2FeCl_3_}*_n_*) is an iron complex with a macromolecular ligand (i.e., a polyazomethine with tetramethyldisiloxane spacer prepared according to the procedure described in already published work [28]. FTIR (KBr) *ν*: 3391 (s), 3221 (s), 2959 (s), 2901 (s), 1668 (s), 1597 (vs), 1508 (s), 1425 (m), 2300 (s), 1258 (vs), 1159 (s), 1059 (vs), 925 (m), 802 (w), 704 (m), 646 (m), 610 (m), 515 (m); GPC: *M*_n_ = 2700, *M*_w_ = 3040, PI = 1.13; Mössbauer spectroscopy: δ = 0.367 mm/s, ΔEQ = 0.649 mm/s, Γ = 0.493 mm/s.

The following reagents were used as received: hexadecylamine (HA), purchased from Sigma-Aldrich, reagent grade, with mp 44 °C, purity 98%; dodecylamine (DA), purchased from Sigma-Aldrich, reagent grade, with mp 28 °C, purity >99%; olive oil (OO), cold pressed (Costa d’Oro, Carrefour, Romania); oleic acid (OA), purchased from Sigma-Aldrich, reagent grade, with mp 14 °C, density 0.89 g/mL, purity >99%; trichloroacetic acid (TCA), purchased from Sigma-Aldrich, ACS reagent grade, purity >99%.

### Equipment

The ultrasonication was performed with an ultrasonic processor, model VC 505, 500 W, 20 kHz. The microwave synthesis was carried out in a sealed vessel and was PC operated (Synergy Software, Discover LabMate, CEM, Inc.) with a monomodal reactor equipped with the IntellyVent pressure controller and magnetic stirring ability. Fourier transform infrared (FTIR) spectra were recorded with a Bruker Vertex 70 FTIR spectrometer in transmission mode with collection of 32 scans, in the range 400–4000 cm^−1^, at room temperature, with a resolution of 2 cm^−1^. Samples were incorporated as dried powder in KBr pellets. Wide angle X-ray diffraction (WAXD) analysis was performed on a Bruker-AXS D8 ADVANCE diffractometer, with a Bragg Brentano parafocusing goniometer. The scans were recorded in step mode using Ni-filtered Cu Kα radiation (λ = 0.1541 nm). The working conditions were 40 kV and 30 mA tube power. The range for recording the spectra was 2θ = 20–100° and 2θ = 20–80° with a time step of 0.5 s/step and 1 s/step. The Bruker computer software packages Eva 11 and Topaz 3.1 were used to plot and process the data. The size of the scan step was 0.01 and 0.02, respectively. For analysis of single crystals and for powder diffraction measurements of several samples, an Oxford-Diffraction XCALIBUR E CCD diffractometer equipped with monochromated Mo Kα radiation was used. Thermogravimetric analysis (TGA) was performed on a Mettler Toledo TGA-SDTA851e type thermogravimetric analyzer under nitrogen flow (20 mL/min), at 10 °C∙min^−1^ heating rate for samples of 2–5 mg each. Alumina crucibles (70 µL) were used as sample holders. Every experiment was repeated three times and showed a good reproducibility. The data were processed using the STAR software from Mettler Toledo. The transmission electron microscopy (TEM) images were taken using a dedicated HITACHI HT7700 microscope operating in high contrast mode at 100 kV accelerating voltage. The samples were prepared by placing small droplets of the diluted dispersion (≈1 g/L) of iron oxide nanoparticles on 300 mesh carbon coated copper grids and dried in vacuum at 50 °C. For qualitative analysis, an energy dispersive X-ray spectrometer (EDX), available in conjunction with the Quanta 200 environmental scanning electron microscope (ESEM), was used. Dynamic light scattering measurements (DLS) were made on a Malvern Zetasizer NS (Malvern Instruments, UK) instrument, which uses noninvasive backscatter detection and a laser wavelength of 633 nm. The Raman spectra were obtained using the Renishaw InVia Refex spectrometer with a 632.8 nm HeNe laser as an excitation source in the spectral region 100–1000 cm^−1^. In order to avoid degradation, the samples were investigated at low incident laser power. The EX-2600X-Calibur SDD (30 μA, 15 kV, 200 s) instrument was used to obtain X-ray fluorescence (XRF) spectra.

### Synthetic procedures

**Thermal decomposition in solution (NPT series).** NPT1a and NPT1b nanoparticles were prepared by introducing a mixture of FeAc1 (0.4 g, 0.63 mmol), dodecylamine (2.0 g, 10.79 mmol), trichloroacetic acid (2.5 g, 15 mmol) and sunflower oil (4 mL) in a three-neck flask placed in a heating mantle and equipped with a condenser, a thermometer, and a glass tube for argon bubbling. The mixture was gradually heated up to 320 °C, kept at this temperature for 30 min, and then gradually cooled. The formed material was washed by dispersing in hexane and filtered to separate larger particles; the filtrate is referred in this paper as NPT1a, while the precipitate as NPT1b. Both samples were redispersed in hexane and ethanol and then centrifuged to remove excess surfactant. NPT2–NPT4 nanoparticles were prepared using a procedure similar to that described for NPT1a, but using the reagents amounts, reaction time and temperature indicated in Table 1.

**Preparation of iron oxide nanoparticles by solvothermal method (NPS series).** For the preparation of samples NPS1–NPS9, reaction mixtures (as indicated in Table 2) were loaded into Teflon autoclaves, which were placed in the hydrothermal stove after sealing. The temperature program consisted of heating at 1 °C/min, up to the maximum temperature shown in Table 2 for each sample, then maintaining this temperature at the maximum for 30 min, followed by cooling at the same rate (1 °C/min). The reaction product was dispersed in hexane and filtered through filter paper. The nanoparticles from the filtrate were precipitated with ethanol and were then centrifuged. To remove excess surfactant, the washing procedure with ethanol and centrifugation were repeated three times.

**Dry thermal decomposition (calcination) (NPC series).** Samples NPC1, NPC2 and NPC3 were prepared by heating of FeAc2, FeCrF and FePAZ, respectively, (0.5 g in each case) in a furnace up to 600 °C in air (heating rate 50 °C/min) and maintained at this temperature for 5 h.

**Preparation of iron oxide nanoparticles under microwave irradiation (NPM series).** FeAc2 (0.3 mmol) was dissolved in 1 mL of distilled water, after which 2 mL of ammonia water (25%) was added; the pH was thus adjusted to 11 for the NPM1 sample and 12 for the NPM2 sample. These solutions were placed in the microwave reactor and irradiated (300 W) at 70 °C for 5 min. The resulting precipitate was washed with distilled water (until pH 7) and centrifuged. The NPM0 sample was prepared using a similar mixture to the one used for NPM1 and NPM2 sample but without microwave irradiation and used as blank sample.

**Preparation of iron oxide nanoparticles under ultrasonication (NPU series).** To prepare the NPU1 and NPU2 samples, 2.0 mmol of FeAc2 were dissolved in 20 mL of distilled water and ultrasonicated for 1 min at 100 °C, then 40 mL of ammonia water (25%) were added to the solution. The ultrasonication time was 10 min for sample NPU1, and 30 min for NPU2.

## Supporting Information

File 1 Additional experimental data.
