# Supplementary material for: From iron coordination compounds to metal oxide nanoparticles
Source: Beilstein J Nanotechnol. 2016 Dec 28;7:2074–87. doi: 10.3762/bjnano.7.198 (PMC5238680; doi:10.3762/bjnano.7.198)
Supplement: File 1 — Additional experimental data. [file Beilstein_J_Nanotechnol-07-2074-s001.pdf]

Supporting Information

for

From iron coordination compounds to metal oxide nanoparticles

Mihail Iacob[1], Carmen Racles[1], Codrin Tugui[1], George Stiubianu[1], Adrian Bele[1], Liviu Sacarescu[1], Daniel Timpu[1] and Maria Cazacu[1]*

Address: [1]Inorganic Polymers Department, “Petru Poni” Institute of Macromolecular Chemistry, Aleea Gr. Ghica Voda 41A, Iasi, 700487, Romania

Email: Maria Cazacu - mcazacu@icmpp.ro

* Corresponding author

Additional experimental data

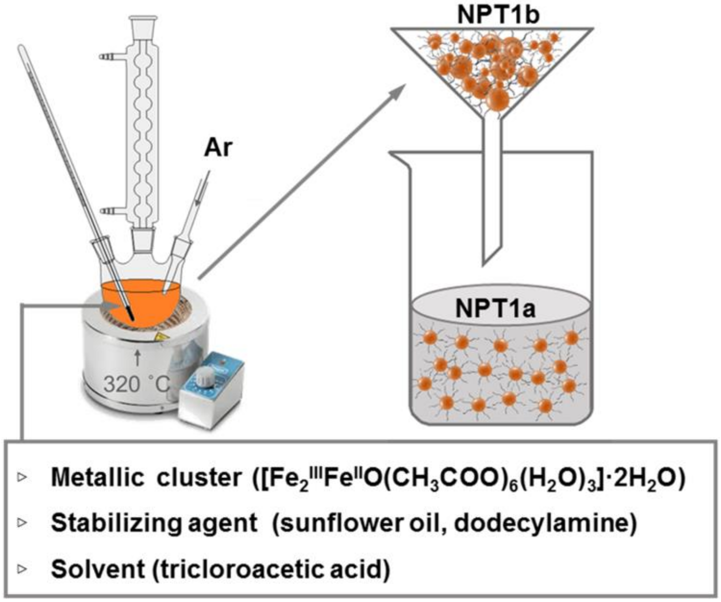

Figure S1: Mixed valence iron acetate conversion into iron oxide nanoparticles by thermal decomposition.

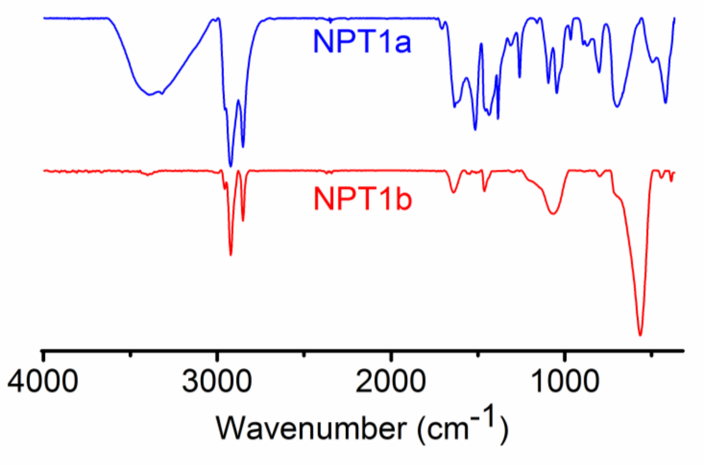

Figure S2. FITR spectra of NPT1 and NPT1b.

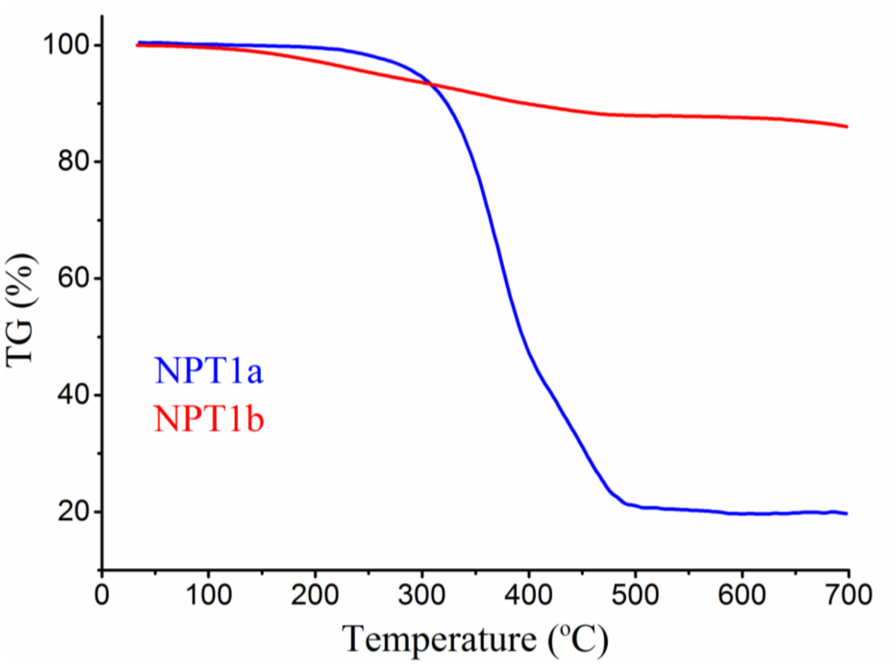

Figure S3. TG curves of NPT1 and NPT1b.

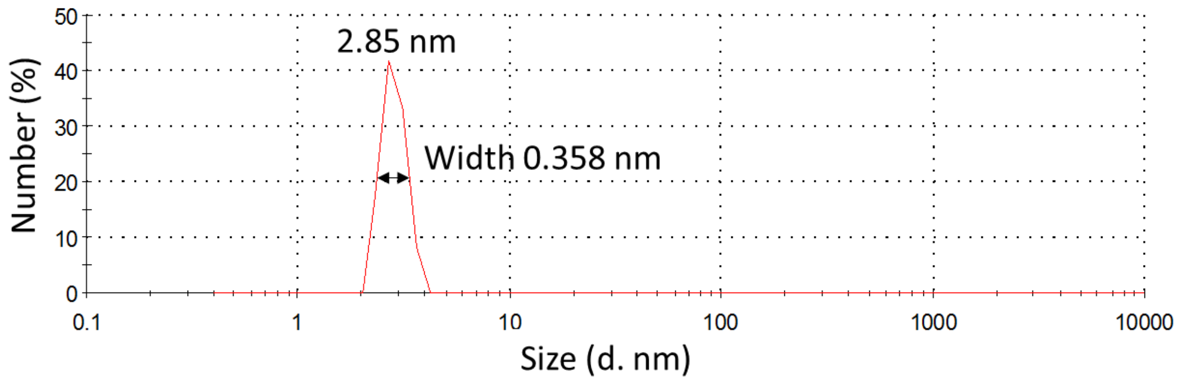

Figure S4. Plot of the hydrodynamic diameter distribution by number of the NPT1a.

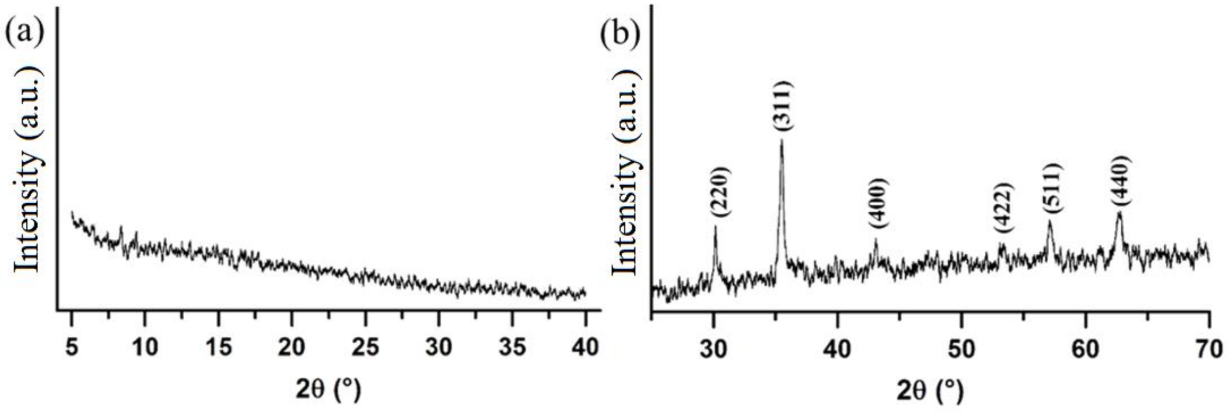

Figure S5. XRD pattern (Cu anode) of NPT1a (a) and NPT1b (b).

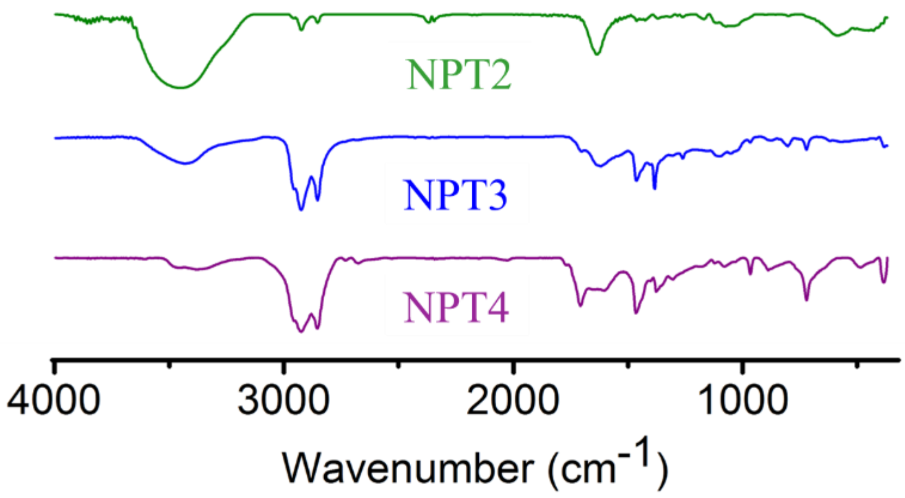

Figure S6. FTIR spectra of NPT2-NPT4.

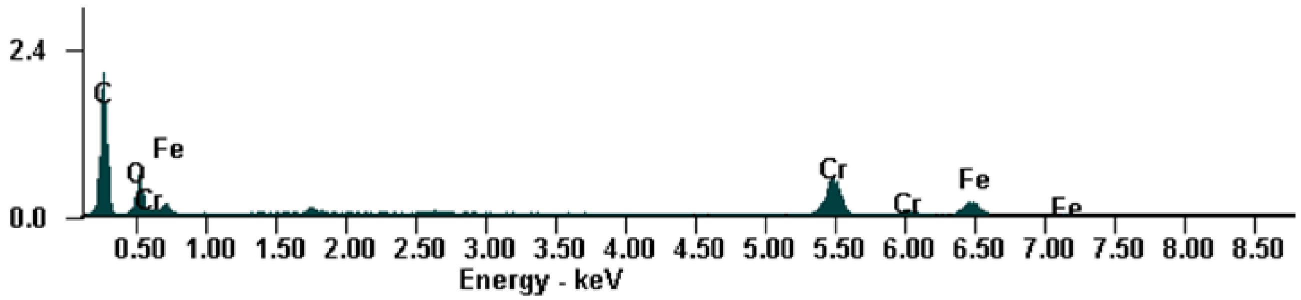

Figure S7. EDX spectrum of NPT4.

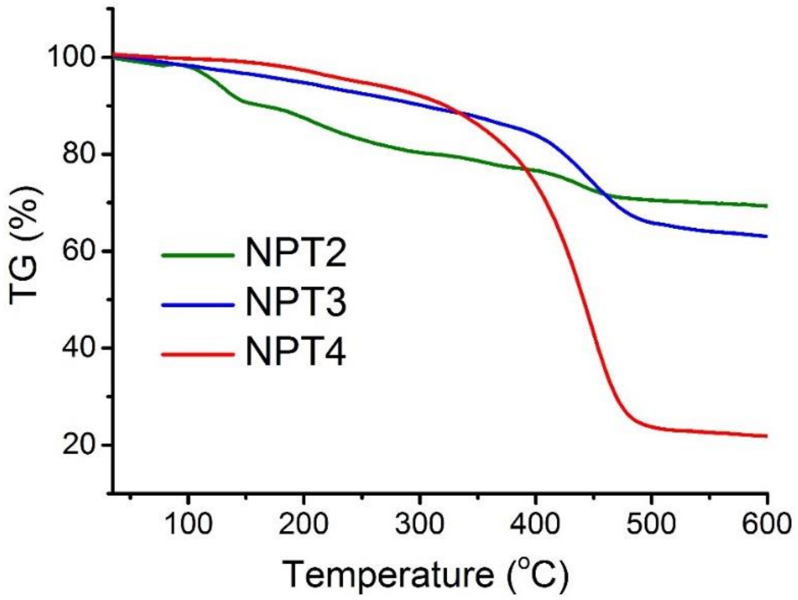

Figure S8. TG curves of NPT2 – NPT4.

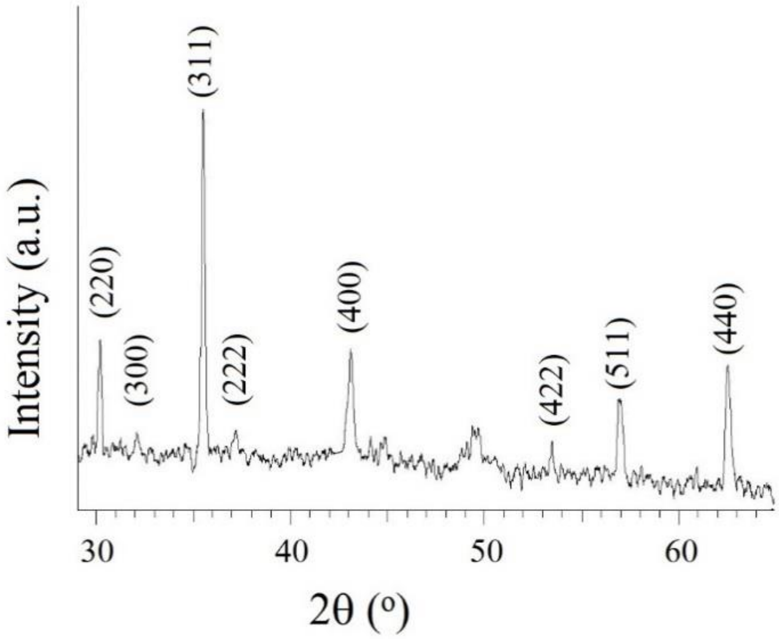

Figure S9. XRD pattern of NPT2.

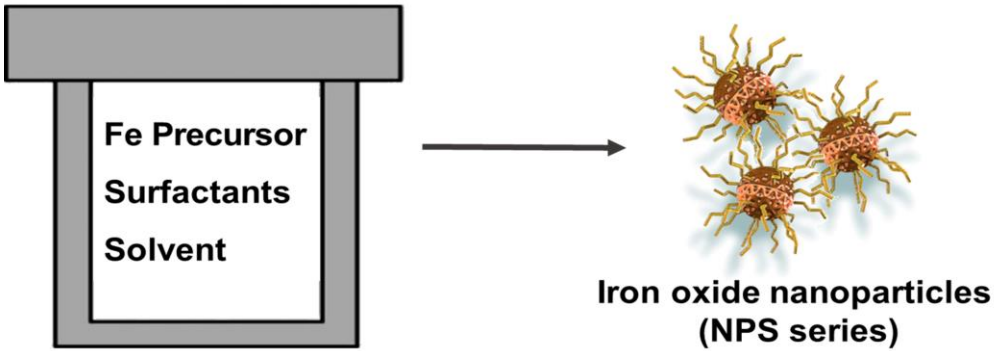

Figure S10. Solvothermal approach for conversion of iron clusters into metal oxides nanoparticles..

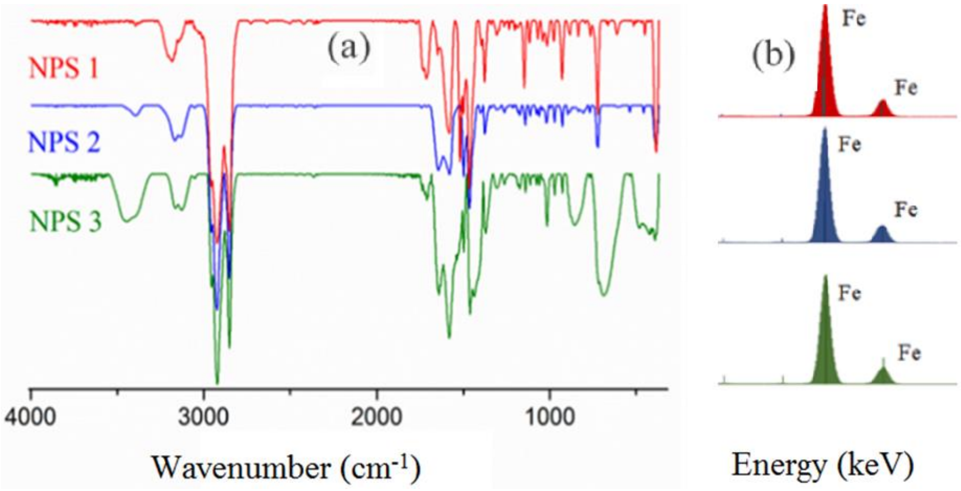

Figure S11. FTIR (a) and XRF spectra (b) of NPS1-3.

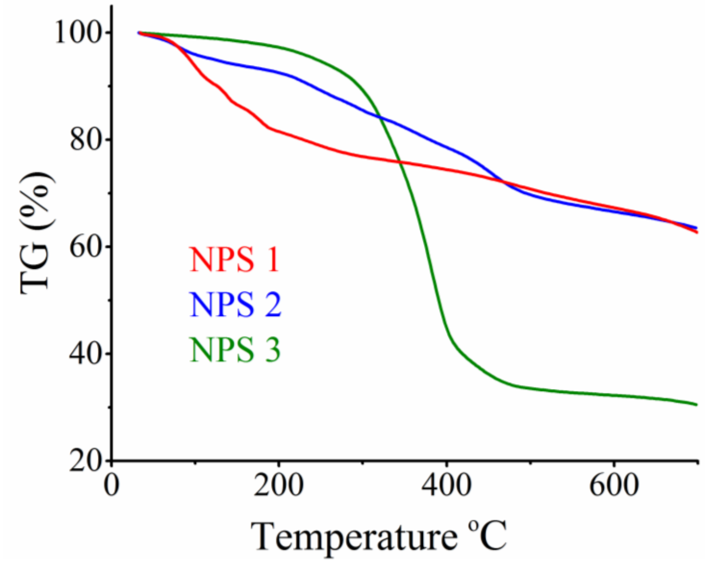

Figure S12. TG curves of samples NPS1-3.

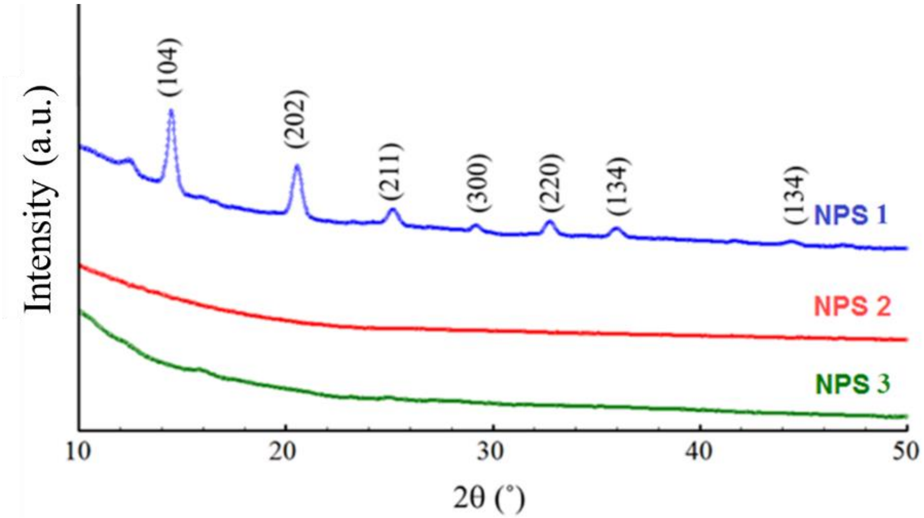

Figure S13. WAXD diffraction patterns of NPS1-3.

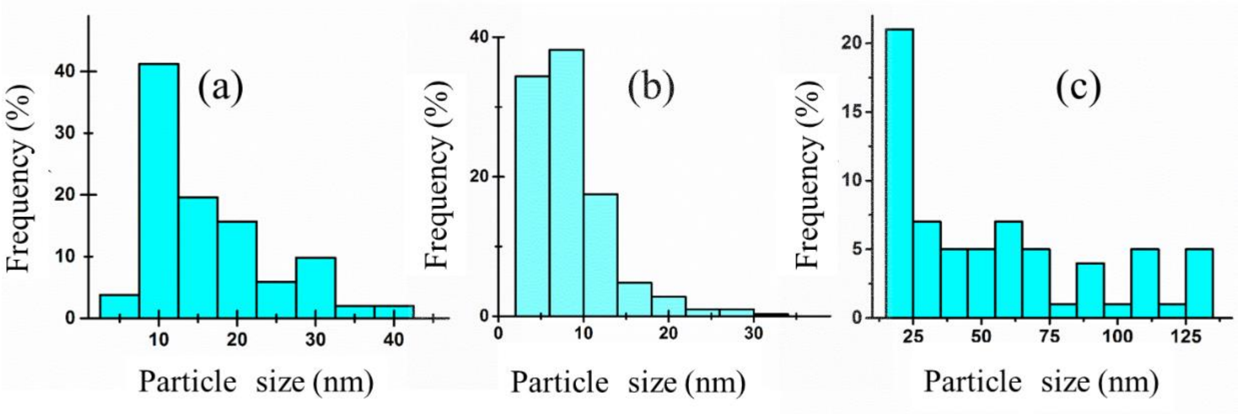

Figure S14. Size distribution histogram of NPS1 (a), NPS2 (b) and NPS3 (c).

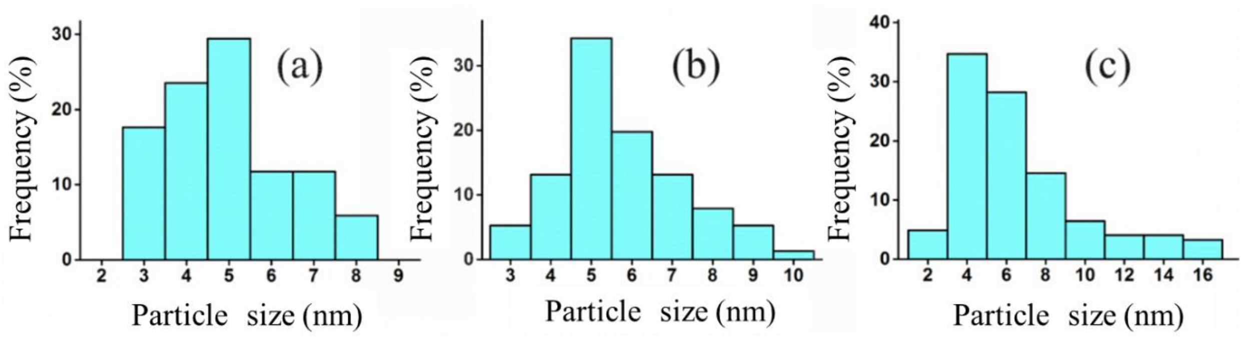

Figure S15. Size distribution histogram of NPS4 (a), NPS5 (b) and NPS6 (c).

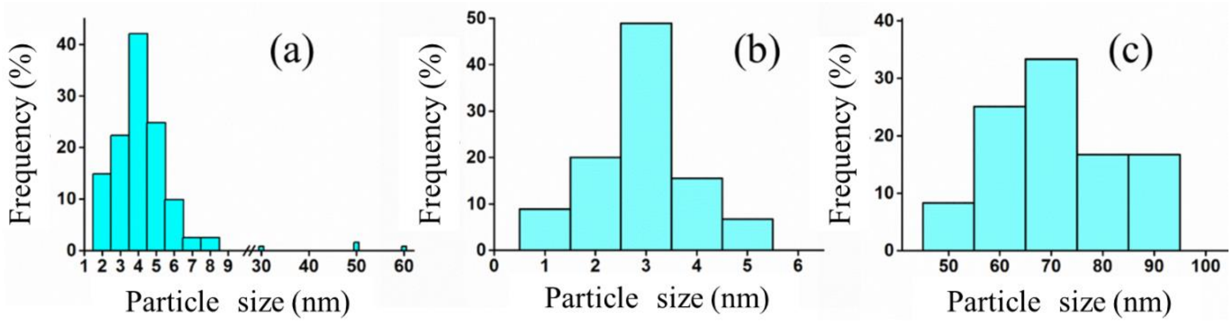

Figure S16. Size distribution histogram of NPS7 (a), NPS8 (b) and NPS9 (c).

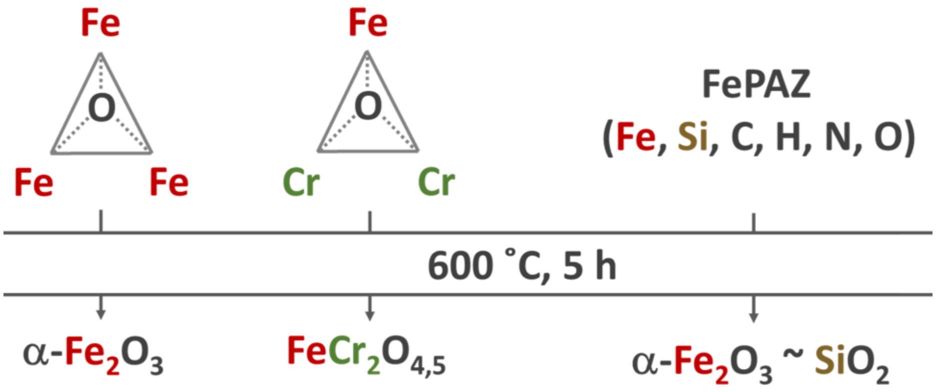

Figure S17. Conversion of iron clusters into iron oxides nanoparticles through calcination.

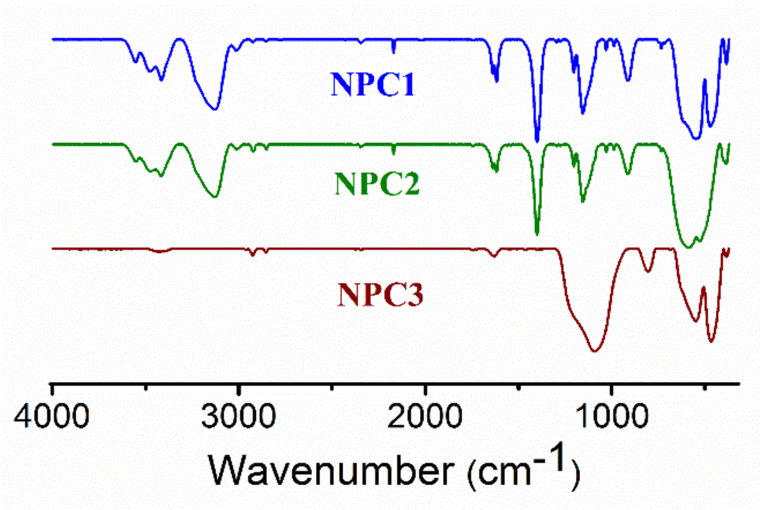

Figure S18. FTIR spectra of NPC1-NPC3.

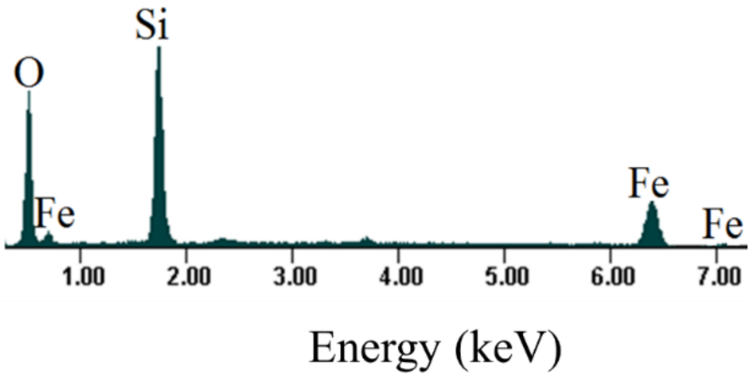

Figure S19. EDX spectrum of NPC3.

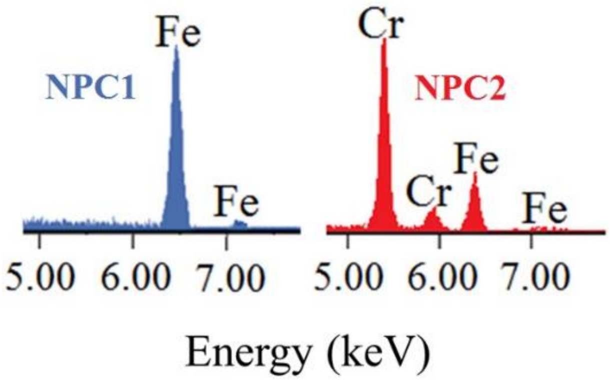

Figure S20. EDX spectra of NPC4 and NPC5.

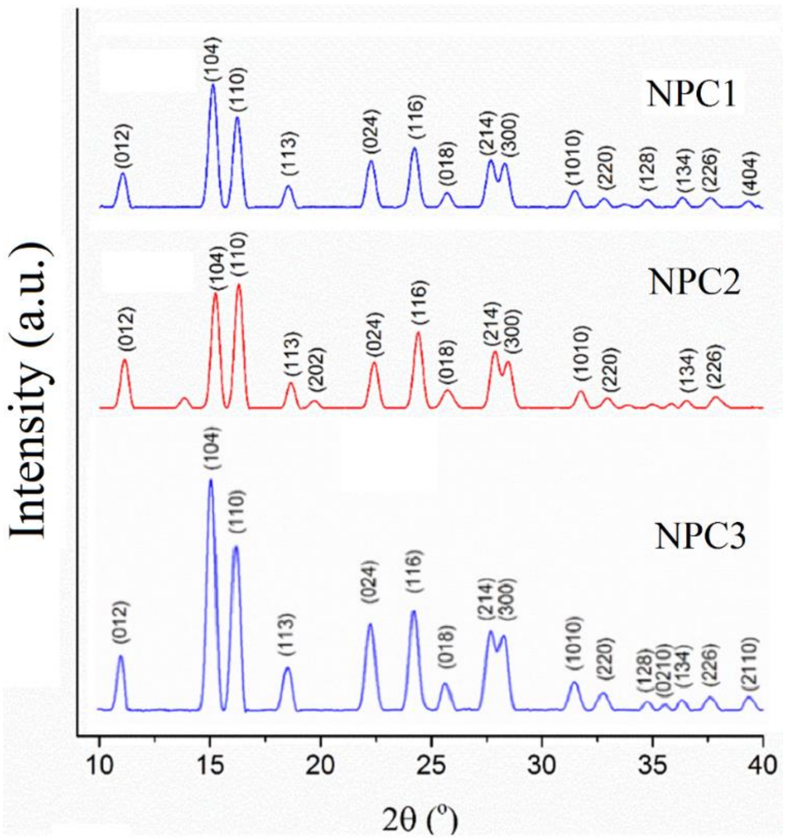

Figure S21. XRD pattern (Mo anode) of NPC1-NPC3.

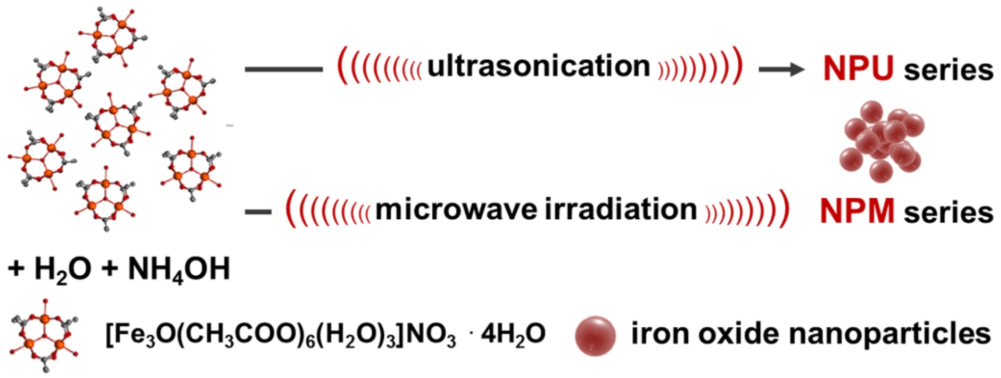

Figure S22. Sonochemical and microwave irradiation approaches for conversion of iron clusters into iron oxides nanoparticles.

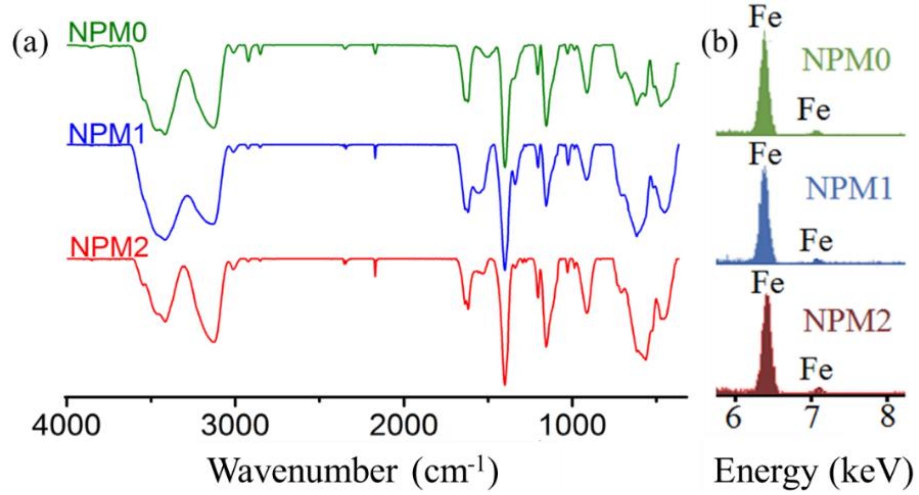

Figure S23. FTIR (a) and EDX (b) spectra of samples NPM0-NPM2.

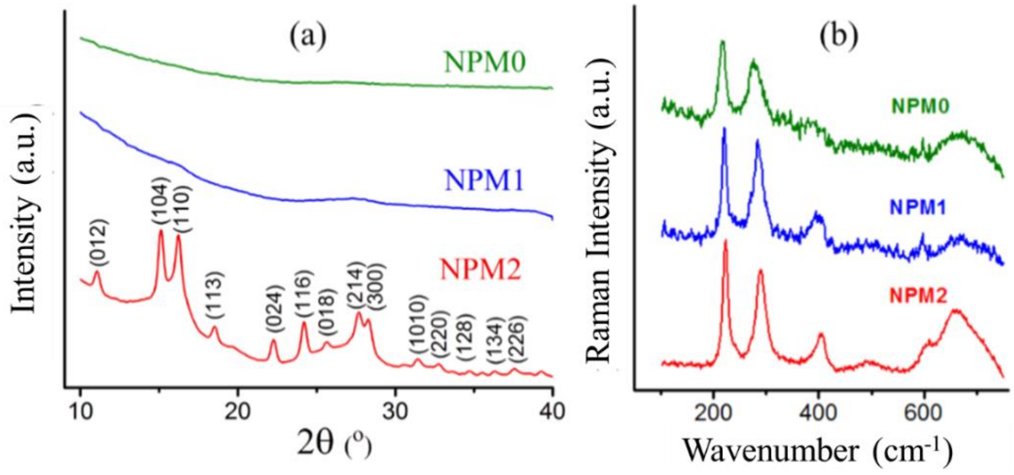

Figure S24. WAXD diffraction patterns (a) and Raman spectra (b) of NPM0-2.

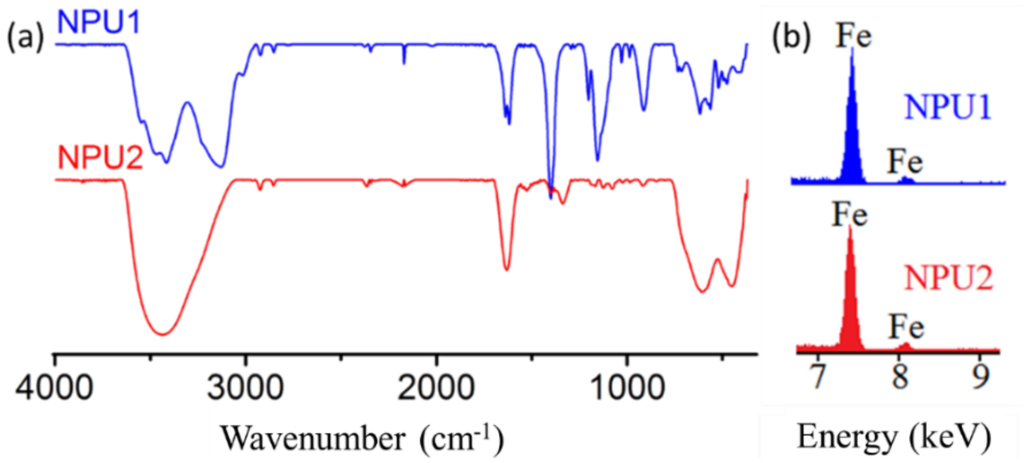

Figure S25. FTIR (a) and EDX spectra (b) of NPU1-2.

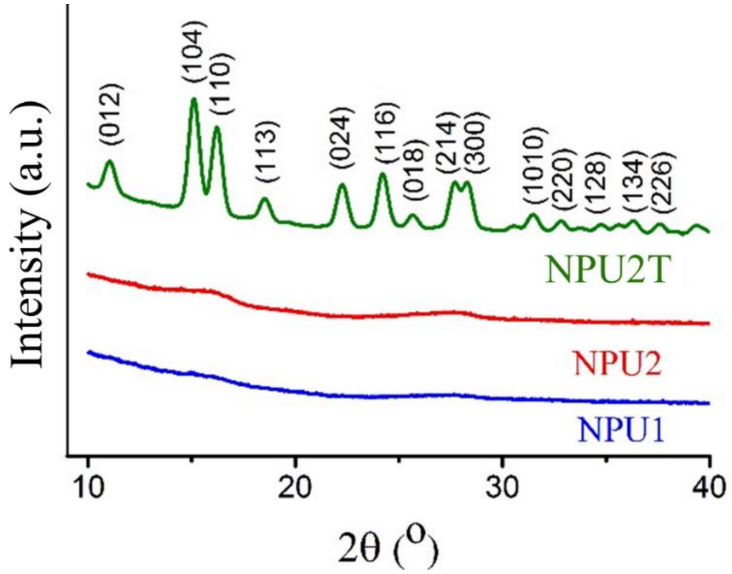

Figure S26. WAX diffraction patterns of NPU1-2 and NPU2T.
